# Patient perspectives on the pathway to psoriatic arthritis diagnosis: results from a web-based survey of patients in the United States

**DOI:** 10.1186/s41927-019-0102-7

**Published:** 2020-01-10

**Authors:** Alexis Ogdie, W. Benjamin Nowell, Eddie Applegate, Kelly Gavigan, Shilpa Venkatachalam, Marie de la Cruz, Emuella Flood, Ethan J. Schwartz, Beverly Romero, Peter Hur

**Affiliations:** 1 Perelman School of Medicine at the University of Pennsylvania, Philadelphia, PA USA; 2 Hospital of the University of Pennsylvania, 3400 Spruce Street, 5 White Building, Philadelphia, PA 19104-4283 USA; 3 Global Healthy Living Foundation, Upper Nyack, NY USA; 4 ICON, Gaithersburg, MD USA; 5 Novartis Pharmaceuticals Corporation, East Hanover, NJ USA

**Keywords:** Psoriatic arthritis, Spondyloarthropathy, Diagnosis, Surveys and questionnaires

## Abstract

**Background:**

There are limited real-world data on the diagnostic experiences of patients with psoriatic arthritis (PsA), including medical care sought and potential barriers to diagnosis. We aim to describe patient experiences related to receiving a PsA diagnosis.

**Methods:**

Ours was a mixed-method, 2-phase study. Phase 1 comprised concept elicitation and cognitive interviews with clinical experts and adults diagnosed with PsA to develop a cross sectional, web-based survey. US adults with a self-reported PsA diagnosis were recruited through a patient support community (CreakyJoints), an online patient research registry (ArthritisPower), and social media outreach. In Phase 2, the online survey collected data on sociodemographics, clinical symptoms, disease burden, and diagnosis history of survey respondents with PsA.

**Results:**

Of the 203 respondents included, 172 (84.7%) were female, and the mean (SD) age was 51.6 (10.8) years. The time between seeking medical attention and receiving a diagnosis was < 6 months for 69 respondents, 6 months to 4 years for 68 respondents, and ≥ 5 years for 66 respondents. Most respondents sought care from general practitioners (79.8%) and rheumatologists (66.5%). Common initial symptoms that led respondents to seek medical attention were joint pain (70.0%) and stiffness (53.7%). Among the initial symptoms that led respondents to seek care, joint pain, swollen joints, and sausage-like fingers or toes (indicating dactylitis) were more common among respondents with shorter time to diagnosis, whereas stiffness, fatigue, enthesitis (indicated by foot problems, tendon and ligament pain), and back pain were more common among respondents with longer time to diagnosis. Common misdiagnoses were psychosomatic issues (26.6%) and osteoarthritis (21.7%). Respondents with shorter times to diagnosis had lower frequencies of misdiagnosis.

**Conclusions:**

Respondents with PsA reported delays in diagnosis and misdiagnoses on their journey to a PsA diagnosis. Symptom differences, such as enthesitis and stiffness, were noted among respondents with shorter vs longer time to diagnosis. Increased understanding of diagnostic barriers may lead to earlier diagnosis and appropriate management to improve outcomes.

## Background

Psoriatic arthritis (PsA) is a chronic inflammatory disease of the skin and musculoskeletal system with an with an estimated incidence of 6 per 100,000 and a prevalence of approximately 1 to 2 per 1000 in the general population [1–3] . PsA is typically characterized by axial skeleton disorders, nail and skin changes, peripheral joint inflammation, enthesitis, and dactylitis, either alone or in combination [4]. This disease is also associated with numerous comorbidities, including hypertension and other cardiovascular conditions, autoimmune diseases, type 2 diabetes mellitus, depression, and chronic obstructive pulmonary disease [5]. Often these comorbidities contribute to the impairment of quality of life, psychological and physical function, and increase in overall clinical burden [6]. Despite advances in treatment, unmet needs still exist in the diagnosis and treatment of PsA [7]. According to the Classification Criteria for Psoriatic Arthritis, a patient must have ≥3 established inflammatory articular diseases of the following: current psoriasis (2 points), personal or family history of psoriasis; current dactylitis or prior history as recorded by a rheumatologist; juxta-articular new bone formation; negative expression of rheumatoid factor; and nail psoriasis, including onycholysis, pitting, and hyperkeratosis [8, 9].

Early diagnosis of PsA is important because a delayed diagnosis significantly contributes to negative patient outcomes [10, 11]. Patients with untreated PsA, even with a 6-month delay from symptom onset to the first rheumatology visit [12], may develop peripheral joint erosions, progressive joint damage, and severe physical limitations [13]. Most patients initially seek medical care from general practitioners [14]; however, patients in consultation with a general practitioner may not receive a timely diagnosis of PsA because it may be challenging for nonrheumatologists to distinguish PsA from other forms of arthritis [15]. Estimates indicate that 6 to 42% of patients with psoriasis eventually develop PsA [16, 17].

There is limited information on the real-world, diagnostic experiences of patients with PsA, including medical care sought and potential barriers to diagnosis. The objective of this study was to determine patient experiences related to the diagnosis of PsA among patients with PsA in the United States.

## Methods

### Data source and study variables

This was a mixed-method, 2-phase study adhering to the Checklist for Reporting Results of Internet E-Surveys (CHERRIES) [18]. Phase 1 consisted of targeted literature review and qualitative interviews with clinical experts and adults diagnosed with PsA via telephone to identify key concepts associated with disease burden and treatment experience (Additional file 1: Table S1 and Additional file 2: Table S2). Verbal informed consent was obtained from all telephone interviewees prior to their participation. The key concepts identified were used to develop an online survey fielded to adults with PsA in Phase 2 (Additional file 3: Table S3). Electronic consent was obtained prior to survey participation. The final questionnaire (Additional file 4: Appendix) consisted of randomized or alternated items to prevent bias, and adaptive questioning was used to reduce the number and complexity of the questions. The questionnaire was composed of 42 questions, with approximately 4–5 questions per page distributed over 10 pages. Only completed responses were analyzed; all questions were enforced, and “not applicable” or “other” responses were provided for certain questions. Respondents were able to review and change their answer through a Back button. By collecting cookies, the Qualtrics platform prevented participants from retaking the survey if they have already completed it. If the participant started the survey, stopped and closed their browser, and then clicked on the email link again sometime later, the participant would be directed back to the page they had left off on, rather than starting the survey over again. As a result, the participants analyzed were unique site visitors. IP address of the client computer was not used to prevent duplicate entries from the same user. An incentive of $25 was provided to participants who completed the entire survey. Only completed questionnaires were analyzed. Participants were required to fill out all questions displayed to them, as the skip logic would not function properly otherwise. The incomplete responses (questionnaires which terminated early) were not analyzed. The average length of time participants spent completing the survey was 27 min. Questionnaires were excluded that were less than 8 min, which was determined by testing the survey and answering questions in a way that would produce the least number of subsequent questions, based on skip logic. The participants were given a unique ID that was passed from the recruitment email to the survey. This ID was used to remove duplicate survey entries. While this was not a “closed” survey, users were prevented from restarting the questionnaire once they had completed it and were directed to the page they had left off if they had paused in the middle of taking it and then resumed. On the chance that a participant completed the survey more than once (for example, by using a different computer), then the unique ID assigned to the user that is passed to the survey from the recruitment email was used to monitor duplicate responses. If a duplicate response was found, the first entry was kept and the most recent was removed from analysis.

Survey participants in the United States aged ≥18 years with a self-reported diagnosis of PsA were recruited for our study via CreakyJoints (https://creakyjoints.org), ArthritisPower (https://arthritispower.creakyjoints.org), and social media outreach in a process similar to a previously reported survey of patients with ankylosing spondylitis [19]. The Global Healthy Living Foundation (GHLF) is the umbrella organization of both CreakyJoints and ArthritisPower, and GHLF investigators identified eligible members based on profile information, which was voluntarily provided, including age, sex, location via zip code, condition, and current medical prescription; no personal information was stored. Our study has been reviewed and approved by a central institutional review board (IRB; Salus IRB). All research was conducted in compliance with the Declaration of Helsinki of 1964 and all later amendments.

This survey workflow went from the recruitment email to the first page of the survey (informed consent form). Of a total of 572 unique email views, the number of respondents who clicked to view the first page of the survey was 326, resulting in a view rate of 57.0%. Of the 326 visitors to the survey site, 258 unique visitors agreed to participate and initiated the survey, resulting in a participation rate of 79.1%. A total of 203 respondents completed the questionnaire for a completion rate of 78.7%. Emails were first sent on September 5, 2017, and the final response was received on October 13, 2017, totaling 38 days of data collection.

### Study variables

Using our web-based survey, respondents provided sociodemographic characteristics (age, sex, race, education, employment, marital status, yearly income, and type of health insurance), clinical information (Routine Assessment of Patient Index Data 3 [RAPID3] cumulative score [1–30], [20–22], RAPID3 categorical disease activity/severity [near remission = 1–3, low = 4–6, medium = 7–12, high = 13–30], extent of psoriasis captured using the Patient Report of Extent of Psoriasis Involvement [23], current symptoms, and other comorbidities), and diagnosis history (time since onset of symptoms, first PsA symptom experienced that prompted the respondent to seek medical help, time between symptom onset and seeking treatment, the types of healthcare providers consulted, time between seeking medical treatment and receipt of formal diagnosis, time since official PsA diagnosis received, and misdiagnoses obtained). Stratification of data was performed based on time between seeking medical attention and receipt of a formal diagnosis of PsA; time cutoffs of < 6 months, 6 months to 4 years, and ≥ 5 years were chosen post hoc to yield an even distribution of the number of respondents across groups.

### Data analysis

Survey results were reported descriptively. Continuous variables were presented using means and SDs, and categorical variables were presented using frequencies and percentages. χ^2^ tests were used to analyze differences across the groups that were stratified post hoc by time to PsA diagnosis; *P* < 0.05 was considered statistically significant and each test between the 3 groups was a unique hypothesis.

## Results

### Sociodemographic and clinical characteristics of respondents with PsA

Among the 203 respondents included in the study, the mean (SD) age was 51.6 (10.8) years; 188 (92.6%) were white, and 172 (84.7%) were female (Table 1). Approximately one-third of respondents reported full-time employment (*n* = 73 [36.0%]), and another one-third were disabled and not working (*n* = 71 [35.0%]). Overall, the time between seeking medical attention and receiving a diagnosis was < 6 months for 69 respondents, 6 months to 4 years for 68 respondents, and ≥ 5 years for 66 respondents. Respondents with faster times to diagnosis (< 6 months) appeared to be slightly older, have undergraduate/postgraduate education, were less likely to be disabled and not working, and were more likely to earn ≥ $100,000/year compared with respondents with longer times to diagnosis.

**Table 1:** Sociodemographic characteristics of respondents with PsA, stratified by time between seeking medical attention and receiving formal diagnosis

Characteristics	Total Respondents (*N* = 203)	< 6 Months (*n* = 69)	6 Months to 4 Years (*n* = 68)	≥ 5 Years (*n* = 66)	*P* Value
Age, years					
Mean (SD)	51.6 (10.8)	53.3 (13.0)	50.6 (9.4)	51.0 (9.6)	0.298
Median (IQR)	51.0 (15.0)	53.0 (16.0)	49.0 (13.5)	50.0 (16.0)	
Range	21.0–82.0	21.0–82.0	31.0–76.0	29.0–78.0	
Female, n (%)	172 (84.7)	56 (81.2)	58 (85.3)	58 (87.9)	–
Race, n (%)					0.052
White	188 (92.6)	59 (85.5)	67 (98.5)	62 (93.9)	
Black/African American	2 (1.0)	0	0	2 (3.0)	
American Indian/Alaska Native	2 (1.0)	2 (2.9)	0	0	
Asian	2 (1.0)	2 (2.9)	0	0	
Multiracial	7 (3.4)	4 (5.8)	1 (1.5)	2 (3.0)	
Prefer not to answer	2 (1.0)	2 (2.9)	0	0	
Highest level of education, n (%)					0.648
High school/GED	10 (4.9)	3 (4.3)	1 (1.5)	6 (9.1)	
Some college (no degree)	47 (23.2)	15 (21.7)	17 (25.0)	15 (22.7)	
Associate’s degree/trade school/certificate program	43 (21.2)	14 (20.3)	15 (22.1)	14 (21.2)	
Undergraduate/postgraduate degree	103 (50.7)	37 (53.6)	35 (51.5)	31 (47.0)	
Current employment status, n (%)^a^					
Employed full time	73 (36.0)	23 (33.3)	24 (35.3)	26 (39.4)	0.769
Employed part time^b^	10 (4.9)	6 (8.7)	4 (5.9)	0	0.042
Disabled/not working	71 (35.0)	21 (30.4)	24 (35.3)	26 (39.4)	0.546
Retired	28 (13.8)	13 (18.8)	8 (11.8)	7 (10.6)	0.363
Other	33 (16.3)	11 (15.9)	13 (19.1)	9 (13.6)	0.704
Relationship status, n (%)					0.349
Married/partnered	137 (67.5)	42 (60.9)	48 (70.6)	47 (71.2)	
Single/separated/divorced	56 (27.6)	21 (30.4)	17 (25.0)	18 (27.3)	
Widowed	10 (4.9)	6 (8.7)	3 (4.4)	1 (1.5)	
Annual income, n (%)					0.117
< $50,000	73 (36.0)	25 (36.2)	25 (36.8)	23 (34.8)	
$50,000 to $99,999	74 (36.5)	18 (26.1)	29 (42.6)	27 (40.9)	
≥ $100,000	41 (20.2)	21 (30.4)	11 (16.2)	9 (13.6)	
Prefer not to answer	15 (7.4)	5 (7.2)	3 (4.4)	7 (10.6)	
Health insurance, n (%)^a^					
Private insurance	132 (65.0)	43 (62.3)	45 (66.2)	44 (66.7)	0.868
Medicare/Medicaid	74 (36.5)	28 (40.6)	25 (36.8)	21 (31.8)	0.562
Other government insurance	10 (4.9)	4 (5.8)	3 (4.4)	3 (4.5)	1.000
Other/don’t know	4 (2.0)	2 (2.9)	1 (1.5)	1 (1.5)	1.000

^a^ Respondents could have selected > 1 option

^b^ *P* < 0.05 comparing respondents with time to PsA diagnosis of < 6 months, 6 months to 4 years, and ≥ 5 years

*GED* general equivalency diploma, *PsA* psoriatic arthritis

More than two-thirds of respondents (68.5%) reported high disease severity as assessed by RAPID3 score (Table 2). Approximately one-fifth of respondents (*n* = 41 [20.2%]) reported moderate to severe extent of psoriasis, assessed by the amount of palm surface area affected. With regard to current symptoms, respondents most often experienced joint pain (86.7%), fatigue (83.3%), and stiffness (78.3%).

**Table 2:** Clinical characteristics of respondents with PsA, stratified by time between seeking medical attention and receiving formal diagnosis

Characteristics	Total Respondents (*N* = 203)	< 6 Months (*n* = 69)	6 Months to 4 Years (*n* = 68)	≥ 5 Years (*n* = 66)	*P* Value
RAPID3 cumulative score (0–30)					
Mean (SD)	14.7 (5.8)	14.3 (6.5)	14.5 (5.8)	15.4 (5.0)	0.559
Median (IQR)	15.5 (8.5)	14.5 (10.2)	15.5 (8.9)	16.3 (6.0)	
Range	1.3–29.0	1.3–29.0	2.0–24.3	2.0–25.2	
RAPID3 disease severity category, n (%)					0.688
Near remission (1–3)	5 (2.5)	2 (2.9)	2 (2.9)	1 (1.5)	
Low (4–6)	14 (6.9)	6 (8.7)	5 (7.4)	3 (4.5)	
Medium (7–12)	45 (22.2)	18 (26.1)	16 (23.5)	11 (16.7)	
High (13–30)	139 (68.5)	43 (62.3)	45 (66.2)	51 (77.3)	
Years since first symptom experienced					
Mean (SD)^a^	15.1 (11.8)	13.4 (13.2)	10.7 (7.6)	21.3 (11.5)	< 0.001
Median (IQR)	12.0 (13.0)	8.0 (13.0)	9.0 (10.0)	18.0 (18.0)	
Range	0–60.0	0–60.0	1.0–39.0	3.0–52.0	
Years since official diagnosis					
Mean (SD)	8.7 (9.6)	10.8 (12.4)	8.1 (7.3)	7.3 (7.9)	0.073
Median (IQR)	6.0 (10.0)	7.0 (11.0)	6.5 (9.0)	5.0 (8.0)	
Range	0–60.0	0–60.0	0–39.0	0–42.0	
Extent of psoriasis (assessed by area covered in number of palms), n (%)^b^					0.259
Little to no psoriasis (< 1 palm)	105 (51.7)	33 (47.8)	43 (63.2)	29 (43.9)	
Only a few patches (1–2 palms)	45 (22.2)	14 (20.3)	12 (17.6)	19 (28.8)	
Scattered patches (3–10 palms)	41 (20.2)	17 (24.6)	9 (13.2)	15 (22.7)	
Extensive psoriasis (> 10 palms)	12 (5.9)	5 (7.2)	4 (5.9)	3 (4.5)	
Current symptoms (in ≥ 50% of respondents), n (%)^c^					
Joint pain	176 (86.7)	57 (82.6)	61 (89.7)	58 (87.9)	0.498
Fatigue	169 (83.3)	56 (81.2)	57 (83.8)	56 (84.8)	0.867
Stiffness	159 (78.3)	53 (76.8)	51 (75.0)	55 (83.3)	0.456
Swollen joints	127 (62.6)	42 (60.9)	45 (66.2)	40 (60.6)	0.759
Reduced range of motion in the joints of the arms and legs	122 (60.1)	43 (62.3)	38 (55.9)	41 (62.1)	0.679
Back pain	119 (58.6)	39 (56.5)	37 (54.4)	43 (65.2)	0.414
Foot problems	115 (56.7)	34 (49.3)	37 (54.4)	44 (66.7)	0.115
Tendon or ligament pain^a^	109 (53.7)	27 (39.1)	37 (54.4)	45 (68.2)	0.003
Difficulty walking	108 (53.2)	39 (56.5)	33 (48.5)	36 (54.5)	0.615
Reduced range of motion in spine and/or hips	107 (52.7)	34 (49.3)	34 (50.0)	39 (59.1)	0.459
Other health conditions (in ≥ 25% of respondents), n (%)^c^					
Depression	126 (62.1)	35 (55.1)	49 (72.1)	39 (59.1)	0.105
Anxiety	118 (58.1)	38 (55.1)	40 (58.8)	40 (60.6)	0.806
Hypertension	97 (47.8)	39 (56.5)	28 (41.2)	30 (45.5)	0.178
Migraines	90 (44.3)	24 (34.8)	34 (50.0)	32 (48.5)	0.142
Fibromyalgia^a^	77 (37.9)	18 (26.1)	30 (44.1)	29 (43.9)	0.043
Irritable bowel syndrome^a^	76 (37.4)	16 (23.2)	26 (38.2)	34 (51.5)	0.003
Seasonal allergies	70 (34.5)	24 (34.8)	20 (29.4)	26 (39.4)	0.464
Dyslipidemia	64 (31.5)	22 (31.9)	19 (27.9)	23 (34.8)	0.689
Gastroesophageal reflux disease	60 (29.6)	22 (31.9)	17 (25.0)	21 (31.8)	0.604
Sleep apnea	53 (26.1)	15 (21.7)	18 (26.5)	20 (30.3)	0.501
Prescription medication ever taken, n (%)^c^					
Biologic	176 (86.7)	–	–	–	–
Nonbiologic DMARD	174 (85.7)	–	–	–	–
Antidepressant	109 (53.7)	–	–	–	–
Steroid medication	162 (79.8)	–	–	–	–
NSAID	167 (82.3)	–	–	–	–
Opioid pain medication	96 (47.3)	–	–	–	–
Other pain medication	132 (65.0)	–	–	–	–
Sleep medication	67 (33.0)	–	–	–	–
Other	58 (28.6)	–	–	–	–
None	3 (1.5)	–	–	–	–

^a^ *P* < 0.05 comparing respondents with time to PsA diagnosis of < 6 months, 6 months to 4 years, and ≥ 5 years

^b^ Extent of psoriasis may be higher due to effects of treatment on reduction of symptom presentation of psoriasis

^c^ Respondents could have selected > 1 option

*DMARD* disease-modifying antirheumatic drug, *NSAID* nonsteroidal anti-inflammatory drug, *PsA* psoriatic arthritis, *RAPID3* Routine Assessment of Patient Index Data 3

### Medical care, diagnosis delay, and misdiagnoses reported by respondents with PsA

**Fig. 1:**
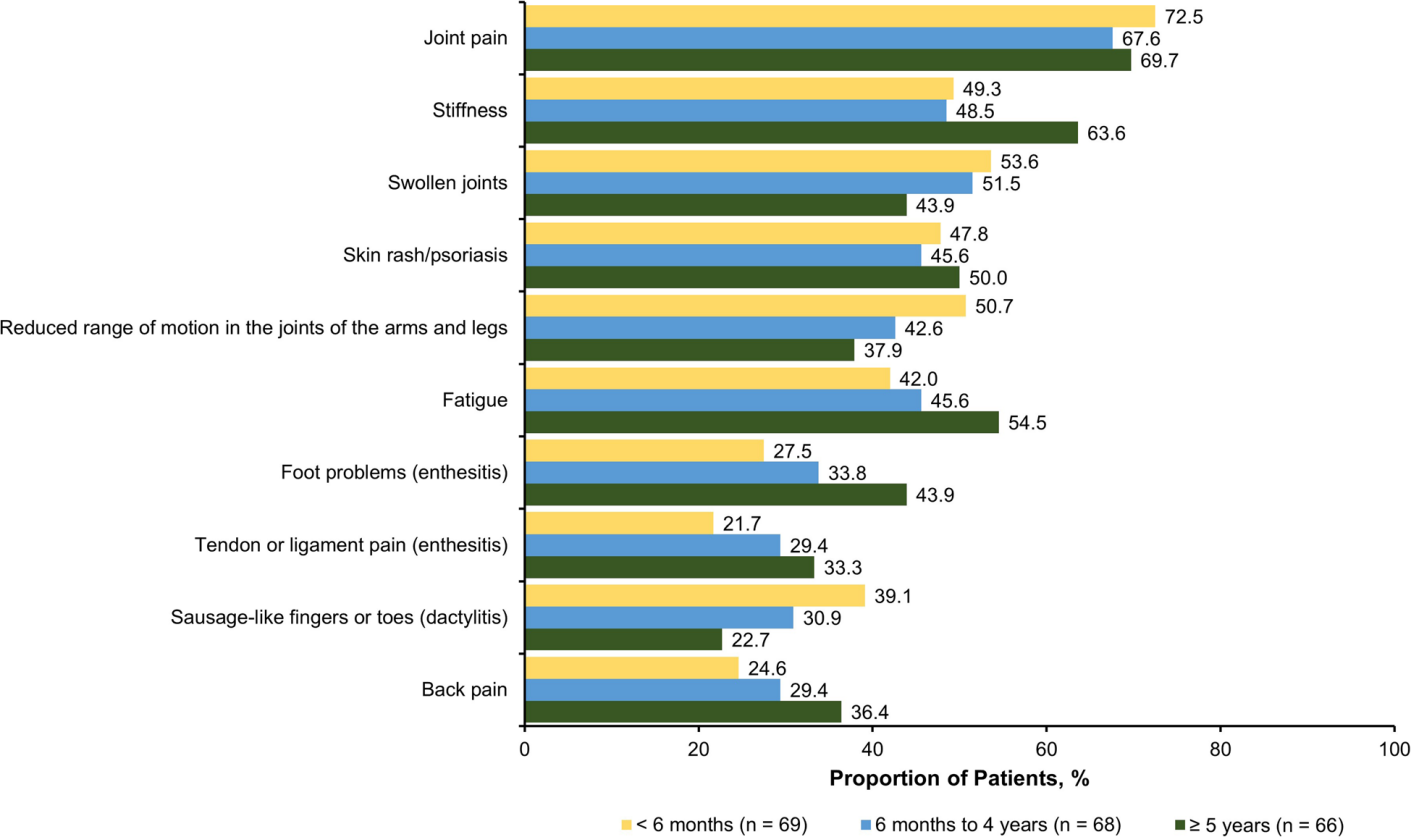
Common first symptoms to prompt seeking health care among respondents with PsA, stratified by time between seeking medical attention and receiving formal diagnosis.* PsA: psoriatic arthritis. * Respondents could have selected > 1 option

**Fig. 2:**
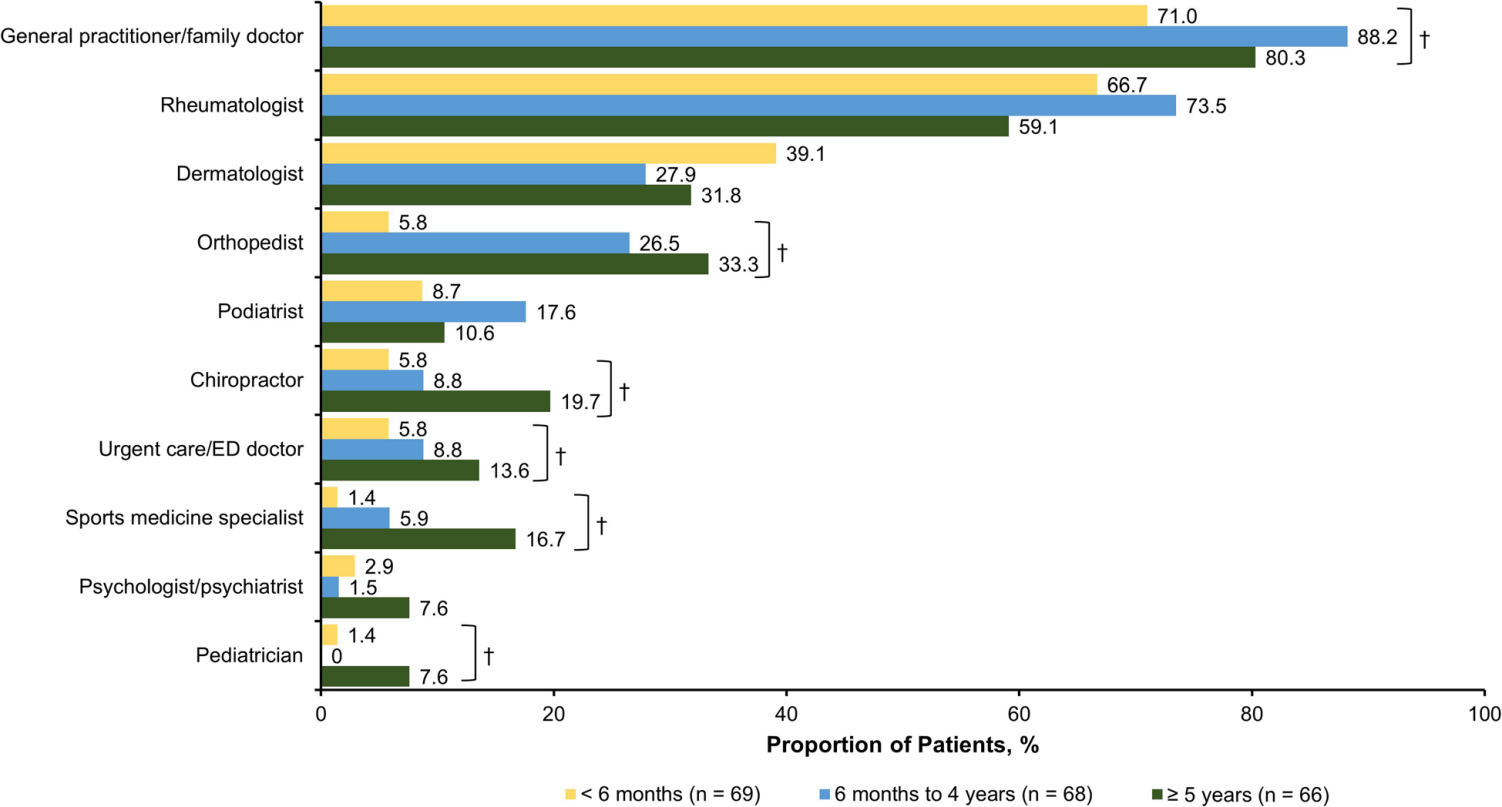
Type(s) of healthcare providers seen during the PsA diagnosis process, stratified by time between seeking medical attention and receiving formal diagnosis.* ED: emergency department; PsA: psoriatic arthritis. * Respondents could have selected > 1 option. ^†^ *P* < 0.05 comparing respondents with time to PsA diagnosis of < 6 months, 6 months to 4 years, and ≥ 5 years

**Fig. 3:**
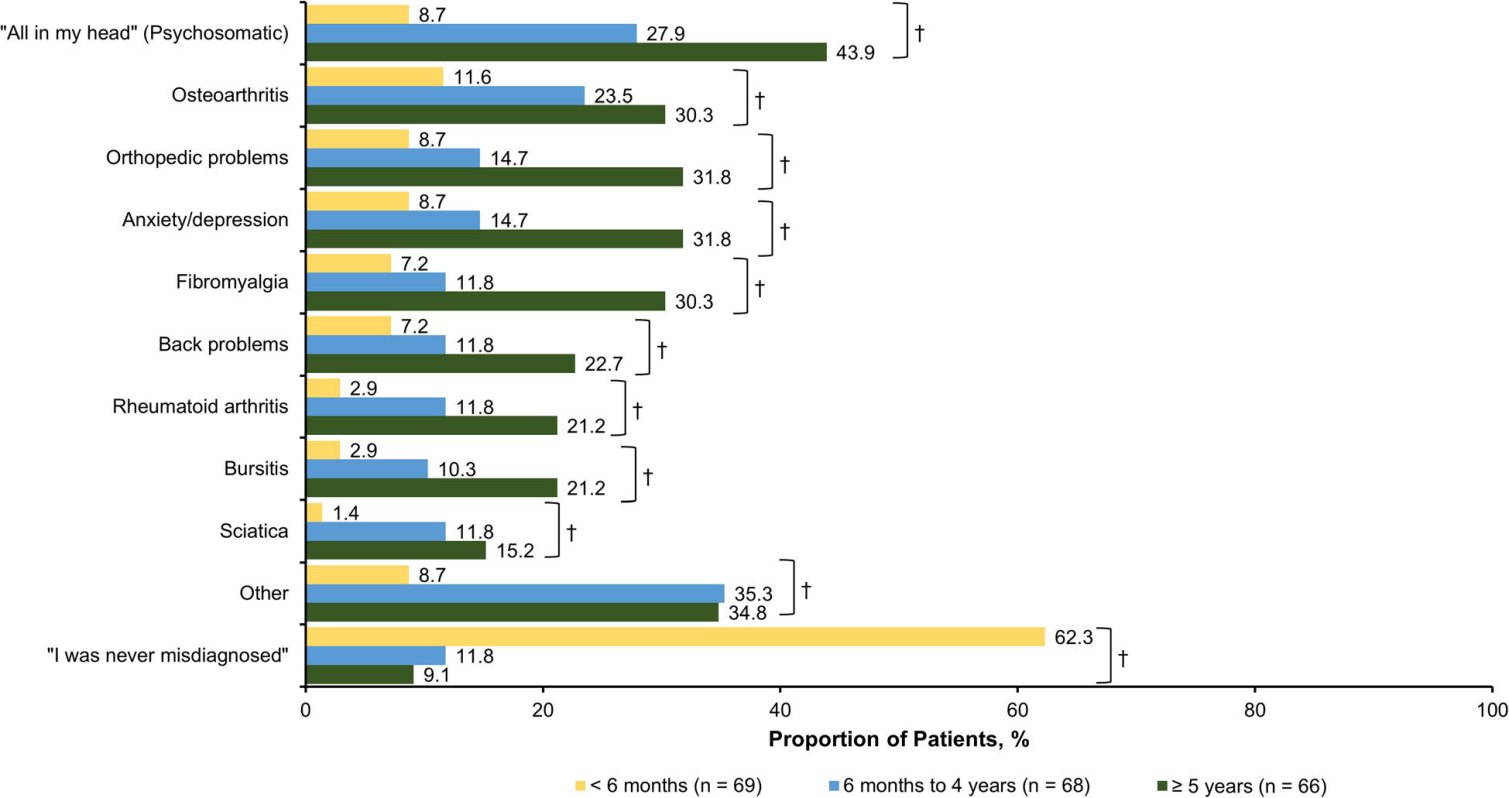
Misdiagnoses received prior to official PsA diagnosis among respondents with PsA, stratified by time between seeking medical attention and receiving formal diagnosis.* PsA: psoriatic arthritis. * Respondents could have selected > 1 option. ^†^ *P* < 0.05 comparing respondents with time to PsA diagnosis of < 6 months, 6 months to 4 years, and ≥ 5 years

The mean (SD) time from symptom onset was 15.1 (11.8) years, and the mean (SD) time since receipt of an official PsA diagnosis was 8.7 (9.6) years. When stratified by time between seeking medical attention and receipt of formal diagnosis (< 6 months, 6 months to 4 years, and ≥ 5 years), the mean (SD) years since first symptom experienced were 13.4 (13.2), 10.7 (7.6), and 21.3 (11.5) years, respectively (*P* < 0.001). With regard to prescription medication, > 80% of respondents had received biologics, conventional synthetic disease-modifying antirheumatic drugs, and/or nonsteroidal anti-inflammatory drugs. The most common symptoms that led to seeking medical care were joint pain (70.0%), stiffness (53.7%), and swollen joints (49.8%) (Fig. 1). Joint pain, swollen joints, reduced range of motion in the joints of arms and legs, and sausage-like fingers or toes (indicating dactylitis) were more prevalent among respondents with shorter time to diagnosis, whereas stiffness, fatigue, foot problems, tendon and ligament pain (indicating enthesitis), and back pain were more common among respondents with longer time to diagnosis. Nearly two-thirds of respondents (65.1%) sought medical treatment within 1 year of symptom onset; 10.3% sought treatment after 1–2 years, and 24.6% sought treatment > 2 years after symptom onset. During their diagnosis journey, respondents reported that they most commonly sought care from a general practitioner (79.8%), rheumatologist (66.5%), dermatologist (33.0%), and/or orthopedist (21.7%) (Fig. 2). Respondents with increasing times to diagnosis (< 6 months, 6 months to 4 years, and ≥ 5 years) were more likely to seek medical consult from general practitioners/family doctors (*P* = 0.043), orthopedists (*P* < 0.001), chiropractors (*P* = 0.033), urgent care/emergency department doctors (*P* = 0.289), and sports medicine specialists (*P* = 0.005), whereas respondents with shorter times to diagnosis tended to seek care from dermatologists (*P* = 0.386) and rheumatologists (*P* = 0.209). The most common misdiagnoses prior to PsA were psychosomatic disorder (26.6%), osteoarthritis (21.7%), anxiety/depression (18.2%), and orthopedic problems (18.2%) (Fig. 3). Only 8 respondents (3.9%) reported that they had never received a misdiagnosis. All misdiagnoses were more prevalent with increasing time to diagnosis.

## Discussion

In this study, we examined the self-reported experiences of US respondents with PsA related to obtaining a diagnosis of PsA. Overall, approximately only one-third of respondents sought medical treatment within 1 year since the onset of symptoms, most of which were musculoskeletal manifestations (eg, joint pain and swelling). Respondents also consulted a variety of healthcare providers, and misdiagnosis prior to receiving the PsA diagnosis was common.

Differences in disease symptoms were observed among respondents with shorter vs longer time to PsA diagnosis. Respondents with shorter time to diagnosis had more occurrences of joint pain, swollen joints, reduced range of motion in the joints of arms and legs, and sausage-like fingers or toes (indicating dactylitis), whereas respondents with longer time to diagnosis had more occurrences of foot problems, neck pain, difficulty walking, uveitis, tendon or ligament pain (indicating enthesitis), and fatigue. These findings suggest that more noticeable symptoms, such as dactylitis and typical joint symptoms, may be more obvious indicators of PsA, leading to a shorter time to diagnosis, whereas less recognized PsA symptoms such as enthesitis, fatigue, and back pain are not as likely to signal a physician to think about PsA. Because a delay in PsA diagnosis contributes to poor outcomes [12], the early recognition of heterogenous PsA symptoms is important for favorable consequences. Patients with delays in diagnosis of PsA have been reported to present with a greater rate of clinical progression and worse physical function compared with patients with an undelayed diagnosis [24–26], and observational studies reported improved outcomes among patients with PsA who were treated soon after receiving a diagnosis of PsA [12, 24, 26]. Improved outcomes after early detection and treatment may be long lasting [25].

Because PsA is often more challenging to diagnose than rheumatoid arthritis [27], a lack of awareness among general practitioners, dermatologists, and primary care physicians can delay the referral of a patient to rheumatologists [28]. Our study revealed that respondents with PsA consulted with several types of healthcare providers during the diagnosis process. In our study, almost 80% of respondents sought medical help from general practitioners and two-thirds from rheumatologists. Another analysis involving 5604 patients with psoriasis or PsA from the National Psoriasis Foundation survey panels reported that of patients who were seeing physicians, 78% consulted with specialists (either a dermatologist or a rheumatologist) and 22% saw internal medicine physicians, family practitioners, or other medical providers [14]. In our study, respondents with increased times to diagnosis were more likely to see general practitioners/family doctors, orthopedists, chiropractors, urgent care/emergency department doctors, and sports medicine specialists, suggesting that they were seeing multiple types of physicians before receiving an accurate PsA diagnosis. Respondents with shorter times to diagnosis were more likely to consult with dermatologists and rheumatologists. Because an estimated 80 to 84% of patients with PsA have psoriasis that precedes arthritis [17, 29], dermatologists are poised for early detection of PsA and rapid referrals to rheumatology. Recommendations for the identification and subsequent referral of patients with inflammatory arthritis have been suggested, including community case searches, public awareness programs, patient education sites on the internet, education programs, self-administered surveys, and referral guidelines for primary care providers [7, 30]. Implementation of such proposals has been shown to positively impact prompt diagnosis [31]. Additionally, validated screening tools to aid in the earlier detection of PsA are also available; some will capture PsA features such as back pain and enthesitis. Examples of PsA screening tools include the Psoriasis Epidemiology Screening Tool [15], Toronto Psoriatic Arthritis Screening (ToPAS) [32], and ToPAS 2 [33]. Results of screening questionnaires administered by healthcare providers in nonrheumatology settings may allow for timely rheumatologist referral and lead to an earlier diagnosis of PsA. Integrated, multidisciplinary clinics involving dermatologists and rheumatologists may substantially increase PsA referral and detection [34]; such settings are available in the United States and in Europe—in Munich, an increase in PsA diagnoses was observed among patients with psoriasis under the primary care of dermatologists when given access to the rheumatology unit [7].

There are limitations to our study, and the findings from this study should be interpreted in the context of limitations inherent to all patient surveys. Respondent perspectives may be subject to recall bias regarding their diagnosis experience. Survey respondents were interfacing within an online community and may have been more likely to participate regularly in research studies and may be more interested in managing their disease. The study relied on self-report of physician diagnosis of PsA; clinician-reported confirmation of diagnosis was not obtained.

## Conclusions

Our study showed that many people living with PsA faced a winding path to arrive at a diagnosis, migrating through various types of healthcare providers prior to the diagnosis. Respondents with shorter times to diagnosis tended to seek care from dermatologists and rheumatologists. Many respondents encountered substantial delays and misdiagnoses before finally receiving a PsA diagnosis. Differences in presenting symptoms may have played a role in time to diagnosis. Symptom differences such as enthesitis and stiffness were observed among respondents with shorter vs longer time to diagnosis. Increased recognition of heterogeneous symptoms associated with PsA, as well as understanding existing diagnostic barriers, may lead to prompt diagnosis and initiation of appropriate treatment that may improve outcomes.

## Supplementary information

**Additional file 1: Table S1.** Summary of the targeted literature review conducted for the identification of key concepts associated with disease burden and treatment experience.

**Additional file 2: Table S2.** Summary table of interview responses with clinical experts with PsA.

**Additional file 3: Table S3.** Concept elicitation from interviews with clinical experts and adults with PsA.

**Additional file 4.** Supplemental Appendix -- Survey.

## Data Availability

The datasets generated and/or analyzed in this study are not publicly available, but are available from the corresponding author on reasonable request.

## Abbreviations

GHLF: Global Healthy Living Foundation
PsA: Psoriatic arthritis
RAPID3: Routine Assessment of Patient Index Data 3
SD: Standard deviation
ToPAS: Toronto Psoriatic Arthritis Screening
US: United States
