# Supplementary material for: Patient perspectives on the pathway to psoriatic arthritis diagnosis: results from a web-based survey of patients in the United States
Source: BMC Rheumatol. 2020 Jan 10;4:2. doi: 10.1186/s41927-019-0102-7 (PMC6953285; doi:10.1186/s41927-019-0102-7)
Supplement: Supplementary file 1 — Additional file 1: Table S1. Summary of the targeted literature review conducted for the identification of key concepts associated with disease burden and treatment experience. [file 41927_2019_102_MOESM1_ESM.docx]

**Supplemental Table 2.** Summary table of interview responses with clinical experts with PsA

|  | **Clinical Expert 1** | **Clinical Expert 2** |
| --- | --- | --- |
| **Background Data** |  |  |
| **Years Treating Psoriatic Arthritis (PsA) Patients** | - 40 years | - 5 years |
| **Average Number of PsA Patients in a Month** | - 30 patients | - 45 patients |
| **Patient Characteristics** |  |  |
| **Age Range of PsA Patients** | - 20s-70s | - 18-80s, peaking in the 40s |
| **Gender Distribution** | - Even male/female distribution | - Even male/female distribution |
| **Race/Ethnicity** | - More common in the Caucasian population; very rare in African-American population | - More common in the Caucasian population than African-American |
| **Socioeconomic Status** | - No trends | - Wide range; higher socioeconomic status patients are more likely to be properly evaluated |
| **Disease Severity Regularly Seen** | - Patients with new onset disease; patients undergoing knee or hip surgery; majority under 50 years | - Mild to severe range; majority in moderate and severe |
| **Defining Disease Severity** | PsA: How many joints are involved; how much psoriasis; and how incapacitated patient is.   - Mild person: Patient probably just starting off with disease - Moderate: Patient has some damage - Severe: Patient has a lot of damage, has had a joint replacement. | PsA: Mild, moderate, severe categorization.   - Mild: Patient has evidence of the disease, but symptoms are not too bothersome or are well-controlled with mild medication such as anti-inflammatories. Patient takes anti-inflammatories and they use topical creams for their psoriasis; no joint destruction. - Moderate: Disease affects quality of life; uses prescriptive therapy. - Severe: Severe impairment of quality of life; more joint destruction from the disease. |
| **Patients Who Seek Optimal Treatment** | - More informed patients - Higher socioeconomic status | - Patients who constantly talk to primary care physician about their pain - Higher socioeconomic status |
| **Other Important Patient Population Characteristics** | - Initially, the presentation of PsA is milder than RA - Many patients initially attribute joint pain to a sports injury and are unaware that it is due to PsA.   “*I think I have the impression, and maybe it’s not the patient population characteristic* *maybe just the disease, that* ***the presentation of psoriatic arthritis is a lot milder initially than the presentation of RA.*** *You know,* ***a lot of these people come in with one joint, they think it’s a sports injury, they don't even know they have psoriasis until you point out certain things to them****. So, I know it’s not what most people think, but I think the psoriatics are a little bit milder in their presentation…”* | - AS population is aging, but medication is not affordable for patients as they age:   *“Um, only that over time the populations age, um, and I think you had mentioned unmet needs earlier, and one of the unmet needs is that* ***while the peak population is relatively young, the amount of people that are 65 and older is getting more*** *and more and more. And one of the unmet needs is that these* ***medications do not, in general, work very well under Medicare****, and so they’re* ***not affordable for these people as they age****.”* |
| **Process of Diagnosis** |  |  |
| **Age at Diagnosis** | - 20 to 40 years of age | - Late 30s, the late 40s, early 40s to early 50s |
| **Typical symptoms/reasons patients seek treatment** | - Unilateral joint swelling - Gout - Achilles tendonitis, tendonitis - Plantar fasciitis - Psoriasis on elbows and knees | - Dactylitis - Psoriasis - Bilateral symmetric rheumatoid-looking arthritis - Spinal pain |
| **Diagnosis Process** | - Patient is seen for unilateral joint swelling. - Patient will usually have subtle psoriasis in their elbows and/or knees. - Labs are ordered: MRI, CRP, sedimentation rate, HLA-B27, as well as a rheumatoid factor and anti-CCP. - Imaging not usually done unless the patient has back pain and back problems and/or they have fairly wide spread peripheral disease   - Otherwise, will order an SI joint X-ray if they’ve had any back pain.   - If X-ray is negative, will do an MRI on the SI joint. | - Look for objective signs of inflammation - Orders labs (sedimentation rate, CRP, rheumatoid factor, CCP) - If patient has back symptoms, an MRI of the SI joints are ordered to get definitive evidence of inflammation - Diagnosis comes down to clinical judgment (psoriatic arthritis vs. osteoarthritis). |
| **Lab Tests/Procedures** | Conduct an exam, plus labs; imaging not as critical:   - CRP - Sedimentation - HLA-B27 - Rheumatoid factor - Anti-CCP. - X-ray - MRI | Conduct an exam, plus labs; imaging is only used if symptoms are felt in the back.   - CRP - Sedimentation rate - CCP - Rheumatoid factor - Anti-CCP. - MRI |
| **Disease Experience and Treatment Course** |  |  |
| **PsA Symptoms** | - Swollen knee or ankle - Achilles tendonitis - Plantar fasciitis | - Fatigue - Joint swelling - Dactylitis - Redness - Joint tenderness - Stiffness - Iritis (10-20%) - Psoriasis (dermatologic) |
| **Symptom Description** | Swollen Joints   - Severity: Enough to interfere with normal activities - Duration: Constant - Variability: Little variation | Swollen Joints   - Severity: Mild to moderate; few severe - Duration: Constant; Worse in the morning, but improves as the day goes on. - Variability: Severity varies daily   Fatigue   - Severity: Mild to moderate; some with severe - Variability: Variable; depends on day, week, month   Iritis   - Severity: Mild to moderate; few severe - Duration: Acute; occasionally can be low-grade and continuous.   Psoriasis (dermatologic):   - Severity: Mild - Duration: Constant - Variability: Changes over many weeks and months |
| **Good Days and Bad Days** | - Little variation during initial phase of disease | - Symptom severity varies daily |
| **Patient Description of Symptoms** | - Pain - Painful knee | - Tired, exhausted - Swollen hands, knuckles, fingers - Stiffness - Iritis: painful, red eye, headache |
| **Most Bothersome for Patients** | - Knee swelling - Ankle swelling | - Joint pain & stiffness |
| **Least Bothersome for Patients** | - None | - Psoriasis (dermatologic) - Iritis due to low proportions on PsA patients with iritis. Those that do develop iritis find it bothersome. |
| **Disease Progression** |  |  |
| **Change in Symptoms Over Time** | - Difficult to assess - Not progressive if treated early; swelling would remain on the same joints that were initially affected - If not treated early, more joints become involved, get recalcitrant to medication leading to greater disability, more pain, less mobility. | - Most patients gradually get worse over time - Some patients remain stable due to unknown reasons - Patients can also “vacillate up and down” due to stress or other factors. |
| **PsA Speed of Progression** | - Progresses very quickly - One case progressed within 3-4 weeks | - Gradual |
| **PsA Variability of Progression** | - Genetics- those who are homozygous carriers may have more joint involvement. | - Most patients gradually get a little worse over time. - Some patients remain more stable for unknown reasons, |
| **PsA Measuring Progression** | - None | - Health assessment questionnaire (HAQ)/RAPID3 - Fatigue: FACIT score, but does not really use it |
| **PsA Tools for Patients to Track Progression** | - None | - Unsure |
| **Patient Burden** |  |  |
| **Daily Impacts** | - Affects work attendance - Affects school activities - Inability to drive - Unable to put on shoes - Mobility issues | - Difficulty performing work tasks - Difficulty with recreational activities - Poor sleep - Difficulties with self-care activities - Difficulty typing - Mobility issues - Iritis- difficulty looking at computer screens |
| **Physical Impacts** | - Influences ability to work - Mobility - Difficulty driving - Difficulty playing sports/doing activities - Difficulty putting on shoes | - Patients can get crippling arthritis in peripheral joints and knees - Difficulty walking - Decreased range of motion - Decreased dexterity - Decreased grip strength. |
| **Social Impacts** | - None | - Female patients report that they do not feel that they can do the activities that they would normally do to socialize (e.g. bridge game). |
| **Work/School Impacts** | - Majority continue to work despite the symptoms - Does not ask patients about missed days | - Missed days |
| **Emotional Impacts** | - Patients report feeling useless | - Depression |
| **Financial Impacts** | - Patients do not usually report financial impacts - Patients mention costs related to medication | - Costs related to treatments |
| **Treatment Pathways** |  |  |
| **PsA Treatment** | - TNF - Encourages exercise | - Topical creams for psoriasis - If mainly peripheral arthritis, patients are given methotrexate or Arava or sulfasalazine, or Otezla   - Used as non-biologic therapies   - Trial period for at least 3 months; Otezla up to 6 months   - Slower to work - If non-biologics are ineffective, patient would then progress to a biologic. - Those with iritis, axial symptoms in the spine, or those with severe PsA initiate with a biologic along with an anti-inflammatory. |
| **Factor(s) Affecting Treatment Decision** | - Patient requests   - If the patient is stable, will either allow patient to stop the medication or may reduce the dosing frequency   - Majority will be on TNF for a long time | - Symptom severity, presence of iritis and/or axial symptoms in the spine. |
| **PsA Patient Involvement om Treatment Decisions** | - Talk to patients about the group of medications and tell them that there are several available in that group - Present pros/cons of each medication, but usually prescribes “drugs that I’ve used over and over and over again and put them on that.” | - Know, understand patients treatment goals and risk factors - Encourages participation and shared-decision making |
| **Treatment Efficacy** | TNFs/Biologics:   - TNFs have been effective - Up until a couple of years ago, TNFs have been the only effective therapies - There are no demonstrable differences in efficacy or safety among TNFs; the main difference is mode and dosing frequency. - TNFs get rid of the underlying cause (inflammatory mediators that are causing the disease) | Anti-inflammatories/NSAIDs:   - Mild to medium efficacy   TNFs/Biologics:   - Moderately to aggressively efficacious |
| **PsA Changing Medications** | - Lack of efficacy - Serious safety event - Otherwise, discourages switching medications. | - If patient does not improve with current regimen or patient is intolerant to the treatment due to side effects, will prescribe a biologic |
| **PsA Changes in Dose** | - Will not change dose; goes by packaging instructions - If the patient is stable may reduce the dose frequency | - If patient is on non-biologic medication and is not experiencing sufficient improvement on lower dosing, will increase the dose to see if that leads to improvement. |
| **Side Effects or Complications of Treatment** | - Infections, specifically upper respiratory infections (i.e. bronchitis, pneumonia) - Urinary tract infections - Cancer | - Anti-inflammatories   - Kidney problems   - Blood pressure   - Swelling   - Heart attack   - Strokes - Biologics   - Infection |
| **Barriers to Treatment** | - Access to care   - Getting into a doctor’s office (delayed diagnosis, high co-pays)   - High costs of medications - Patient delays seeking care; assumes symptoms are normal - Treatment barriers- patients are hesitant to undergo therapy due to side effects. | - Medication costs, especially biologics and non-biologics   - Cimzia and Remicade are the only two medications for ankylosing spondylitis and psoriatic arthritis that work under Medicare’s medical benefit   - Newer treatments have to go on Medicare Part D plans, which make these medications unaffordable. - Costs of labs - Distance to physician, especially for those in rural communities. |
| **PsA Symptom Improvement** | - Joint swelling - Bursitis | - Psoriasis (dermatologic) - Joint pain - Joint swelling - Stiffness |
| **PsA Symptoms Unlikely to Improve with Treatment** | - Nail changes - Dactylitis | - Fatigue |
| **PsA Treatment Effects You (the Doctor) Look For** | - Reduction in frequency of joint swelling; severity can stay constant - Ability to return to normal activities | - Effective treatment that does not increase risk of infections - Treatment that is affordable - Treatment that is available for all types of insurance - More effective - Treatment with a different mechanisms of action (affects immune system in a different way) |
| **PsA Treatment Effects Patients Look For** | - Reduction in joint swelling - Ability to return to normal activities | - Improving signs and symptoms - Affordable treatment |
| **PsA Unmet Needs** | - Primary care physicians (internists, family practitioners) need better education and understanding of the condition. | - Medications are not affordable for patients as they age; limited Medicare coverage - Laboratory work is costly - Those in rural communities have to travel to get laboratory work. - Lack of awareness in patients. |
| **Anything Else?** | - Health care professionals continue to prescribe methotrexate despite limited evidence of efficacy in AS & PsA | - Nothing else |

## 
