# Supplementary material for: Patient perspectives on the pathway to psoriatic arthritis diagnosis: results from a web-based survey of patients in the United States
Source: BMC Rheumatol. 2020 Jan 10;4:2. doi: 10.1186/s41927-019-0102-7 (PMC6953285; doi:10.1186/s41927-019-0102-7)
Supplement: Supplementary file 2 — Additional file 2: Table S2. Summary table of interview responses with clinical experts with PsA. [file 41927_2019_102_MOESM2_ESM.docx]

| **Supplemental Table 1.** Summary of the targeted literature review conducted for the identification of key concepts associated with disease burden and treatment experience | | | | |
| --- | --- | --- | --- | --- |
| **Literature search:**  1. ((psoria* adj3 arth*) or (psoriatic adj2 (polyarthritis or rheum*)) or PsA).mp.  2. (unmet adj1 need$).ti,ab,kw.  3. (disease adj1 burden$).ti,kw,ab.  4. (treatment adj1 concern$).ti,kw,ab.  5. (treatment adj1 burden).ti,kw,ab.  6. (treatment adj1 challenge$).ti,kw,ab.  7. qualitative research/  8. (focus adj1 group$).ab,ti,kw.  9. interview$.ti,ab,kw.  10. *quality of life/  11. *questionnaire/  12. *outcomes research/  13. *rating scale/  14. *functional assessment/  15. (quality of life or QOL or health related quality of life or HRQOL or HRQL).ti,ab.  16. Exp interview/  17. Focus group.tw.  18. Or/2-17  19. 1 and 18  20. Limit 19 to (human and English language and (adult<18 to 64 years> or aged <65+ years>) and last 5 years) | | | **Eligibility Criteria:**   - Adults only (18 yrs+) - Published within the last 5 years - English language - Human studies - No case studies - Minimum of N=10 patients - Prioritize qualitative studies that describe impact of the disease and treatment on the patient including impact on quality of life and treatment challenges/access to treatment - No instrument development papers unless focused on qualitative work | |
| **Source** | **Study Objective / Design** | **Sample Characteristics** | **Relevant Results** | **Symptoms/Impacts/Unmet Needs/Access to Care** |
| Armstrong *et al.,* 2012 | **Objective:** To ascertain impairment in quality of life and work productivity among patients with psoriasis and psoriatic arthritis.  **Design:** retrospective  **Timeframe:** 2003-2011  **Methods:**  Survey data were collected from psoriasis and psoriatic arthritis patients in the general community in the U.S. Data from 13 cycles of surveys (2003–2009, 2011), which were collected from a database consisting of more than 75,000 patients. During each survey cycle, over 400 participants were identified through random sampling. | **Condition**: PsO and PsA  **Country:** USA  **Characteristics**:   - The combined survey cycles yielded 5,604 participants who completed the surveys. - N=1286 with “mild” psoriasis (61% female, 88% white), N=2031 with “moderate” psoriasis (60% female, 88% white), and N=1894 with “severe” psoriasis (60% female, 85% white) - N=359 (28%) with “mild” psoriatic arthritis, N=637 (31%) with “moderate” psoriatic arthritis, N=879 (46%) with “severe” psoriatic arthritis   **Inclusion criteria:**   - Adults (>18 years of age) with psoriasis alone and comorbid PsA   **Exclusion criteria:**   - n/a | **Key Results:**   - Psoriasis and psoriatic arthritis affected overall emotional wellbeing in 88% of patients, and they interfered with enjoyment of life in 82%. - Most patients reported experiencing anger (89%), frustration (89%), helplessness (87%), embarrassment (87%), and self-consciousness (89%). - Many patients actively concealed physical manifestations of their diseases (83%), and experienced pain (83%) and pruritus (93%) regularly. - Of note, 12% of patients were unemployed, and 11% worked part-time. Among unemployed patients, 92% cited psoriasis and/or psoriatic arthritis as the sole reasons for not working. Among working patients, 49% missed work days regularly due to psoriasis. Compared to patients with mild psoriasis, patients with severe psoriasis have 1.8 times greater odds to be unemployed after adjusting for age and gender (Adjusted OR = 1.7, 95% CI 1.4–2.3). | **Key Symptoms:**   - Itchiness - Physical irritation - Physical pain or soreness - Disfigurement   **Key Impacts:**   - QoL - Emotional wellbeing - Self-consciousness - Unsightly appearance - Anger or frustration - Helplessness - Embarrassment - Concealment with clothing - Try to conceal physical manifestations of disease - Work absenteeism/presenteeism |
| Chisholm *et al*., 2016 | **Objective:** To explore people’s illness beliefs, emotions and behaviors that relate to living with PsA and account for the distress experienced by those living with this condition.  **Design:** Qualitative, semi-structured interviews  **Methods:**   - Semi-structured individual interviews were conducted in the UK with adults with PsA. - Interview questions theoretically informed by Common-Sense Self-Regulation Model (CS-SRM). - Two stages of data analysis, in line with thematic and framework analysis principles, involved coding pre-determined CS-SRM components and identifying any additional emergent themes. - Constant comparison techniques allowed for patterns across CS-SRM components to emerge inductively from the data. | **Condition**: PsA  **Country:** UK  **Characteristics**:   - Twenty-four people with PsA participated (54% male; aged 27-71; time since onset ranged between 4 months and 29 years; 21 white British (87.5%) and 3Asian (12.5%)).   **Inclusion criteria:**   - Adults (>18 years old) diagnosed with PsA (three or more swollen and three or more tender joints in line with current recommendations and ClASsification of Psoriatic ARthritis (CASPAR) criteria.   **Exclusion criteria:**   - n/a | **Results:**   - Patients reported consequences of living with PsA that affected all areas of their social, work and family lives but most prominently described how physically restricting PsA was. Patients feared their condition would rapidly and unexpectedly deteriorate, resulting in dramatic loss of functioning. - Patients described how PsA contributed to limiting their past/current life course and decisions. PsA had prevented romantic relationships, starting a family, engaging in school, developing desired careers, learning to drive, or moving house. The profound consequences and restrictions reported were often accompanied by feelings of helplessness and hopelessness. Patients said they were unable to visualize a future for themselves and often expressed suicidal ideation. For some patients this related to the impact of PsA upon their lives (e.g. damaged marriage/family life) and for others it was directly related to PsA symptoms (e.g. worsening/permanence of pain). - Patients acknowledged instances in which significant others (i.e. family members/colleagues) offered physical and emotional support while in contrast feeling that people do not take their condition seriously. They reported anger and low mood from being dismissed or disbelieved by members of the public, family or friends and health care staff. - Some positive experiences in healthcare settings were reported, particularly in relation to being referred to experts in PsA, and receiving life-changing pharmacological treatments. However, some described early dismissal of symptoms in non-specialist settings, healthcare professionals being uninterested in the impact of PsA on their lives, their concerns not being taken seriously and being made to feel like an inconvenience. - Patients felt the healthcare system had failed to prepare them for living with PsA and the influence it would have in their lives, specifically that existing patient resources were poor at explaining the physical and psychological factors associated with PsA. Patients expected more support from the healthcare system to manage life with PsA or to identify symptoms earlier to enable a more prompt diagnosis. - Throughout patients’ accounts of living with PsA, they expressed a sense of injustice, describing ways in which PsA threatened their personal identity and making negative comparisons with other people. - In managing day-to-day, patients described attempts to push through, hide or avoid aspects of their condition. In line with this, they frequently reported that they simply put up with the symptoms and consequences of PsA. Patients engaged in internal brain-vs-body conflicts about whether to be active or rest; these battles were resource-intensive causing fatigue and distress. | **Key Symptoms:**   - Fatigue   **Key Impacts:**   - Social life - Work - Family life - Physical restrictions - Fear of disease progression - Romance/sex life - Participation/willingness to participate - Driving - Psychological/emotional effects - Hopelessness - Dismissal by society and HCPs - Family blame/judgment - Premature ageing   **Unmet Needs:**   - Misperceptions/education - Support programs (i.e. ineffective coping styles) - Insufficient societal support/understanding - Occasional early dismissal of symptoms in non-specialist setting - Improperly prepared by healthcare system to live with PsA - Existing patient resources poor at explaining the physical and psychological factors associated with PsA - Managing life with PsA   **Access to Treatment/Care:**   - Misperceptions, ineffective coping styles (e.g., avoidance/blocking) and negative emotions should be actively identified and addressed with people with PsA. - Patients felt they were referred to the correct experts in PsA and received life-changing pharmacological treatments. - Some patients reported early dismissal of symptoms in non-specialist settings. - Some patients reported feeling that their HCPs were uninterested in the impact of PsA on their lives. |
| Edson-Heredia *et al.,* 2015 | **Objective:** To compare disease characteristics, comorbidities, and psoriasis-related QoL in patients with moderate to severe psoriasis with and without comorbid PsA.  **Design:** retrospective  **Methods:**   - Since 2003, NPF has conducted semiannual survey panels to collect patient-reported data on topics including disease characteristics, treatment utilization, and psoriasis-related QOL impact. - Responses from approximately 5604 individuals have been collected and analyzed from a total of 13 survey panels conducted from 2003-2009 and 2011. - In each survey panel, responses from approximately 400 randomly selected respondents were collected, with approximately 300 respondents having moderate to severe psoriasis and 200 respondents being NPF members. | **Condition:** PsO with/without PsA  **Country:** USA  **Characteristics:**   - Of 5467 total respondents, 3532 reported moderate to severe psoriasis of which 1280 (36.2%) had comorbid PsA. - Compared to patients with moderate to severe psoriasis alone, those with moderate to severe psoriasis and comorbid PsA were older, less commonly male, less likely to be employed, had a longer duration of psoriasis, were more likely to be white, and were more likely to have health insurance coverage. - Among the group with PsA, patients with moderate to severe psoriasis were more likely to be treated by a dermatologist only versus those with mild or no psoriasis.   **Inclusion criteria:**   - Adults (>18 years of age) with psoriasis alone and comorbid PsA   **Exclusion criteria:**   - n/a | **Results:**   - Patients with moderate to severe psoriasis and comorbid PsA reported significantly higher rates of diabetes mellitus, lupus, rheumatoid arthritis, other arthritis, ankylosing spondylitis, and high blood pressure than patients with moderate to severe psoriasis alone (p<.05) - In the group with PsA, patients with moderate to severe psoriasis were significantly more likely to report diagnoses of colitis, diabetes mellitus, heart disease, and high blood pressure compared to those with mild to no psoriasis - Patients with moderate to severe psoriasis and comorbid PsA reported a greater impact on overall QOL compared to their counterparts with moderate to severe psoriasis alone. Similarly, patients with moderate to severe psoriasis and comorbid PsA reported a greater impact of itching, physical irritation/soreness, and pain symptoms. - In the PsA group, patients with moderate to severe psoriasis also reported significantly worse itching, irritation, pain, and overall QOL compared to their counterparts with mild to no psoriasis (p<.05). | **Key Symptoms:**   - Itching/irritation - Pain - Fatigue   **Common comorbidities**   - Ankylosing spondylitis - Colitis - Crohn’s disease - Diabetes mellitus - Heart disease - High blood pressure - High cholesterol - Lupus - Multiple sclerosis - Rheumatoid arthritis - Other arthritis   **Key Impacts:**   - Physical impairment   **Unmet Needs:**   - Managing disease severity - Need to better recognize/screen for PsA in patients with psoriasis   **Access to Treatment/Care:**   - Highlight the need to screen for PsA in patients with psoriasis and effective treatment of skin disease in patients with PsA |
| Feldman *et al.,* 2014 | **Objective:** To compare the prevalence of comorbidities, health care utilization, and costs between moderate-to-severe psoriasis (PsO) patients with comorbid psoriatic arthritis (PsA) and matched controls.  **Design:** retrospective  **Timeframe:** 2007-2012  **Methods:**   - Adults ages 18–64 years with concomitant diagnoses of PsO and PsA (PsO+PsA) were identified in the OptumHealth Reporting and Insights claims database between January 2007 and March 2012. - Moderate-to-severe PsO was defined based on the use of at least one systemic or phototherapy during the 12-month study period after the index date (randomly selected date after the first PsO diagnosis). - Control patients without PsO and PsA were demographically matched 1:1 with PsO1PsA patients. Multivariate regressions were employed to examine PsO/PsA-related comorbidities, medications, health care utilization, and costs between PsO+PsA patients and controls, adjusting for demographics, index year, insurance type, and non–PsO/PsA-related comorbidities. | **Condition:** PsO+PsA  **Country:** USA  **Characteristics:**   - Both cohorts had a mean age of 48.5 years and 47.9% were women**.** - More than 30% of the sample lived in the South, followed by the Midwest and Northeast regions. Approximately two-thirds of the matched pairs had insurance coverage from a preferred provider organization. - Compared with matched controls, PsO1PsA patients had a higher mean modified CCI score (0.23 versus 0.12; P , 0.0001), and a greater proportion of them had non–PsO/PsA-related comorbidities. Specifically, the PsO+PsA group had a significantly higher prevalence of lung disease (9.5% versus 6.1%), liver disease (3.4% versus 1.0%), and rheumatic disease unrelated to PsO/PsA (1.1% versus 0.2%) (all P , 0.05).   **Inclusion criteria:**   - Patients in the PsO cohort had at least 2 diagnoses of PsO (ICD-9-CM code 696.1) on different dates between January 1, 2007 and March 31, 2012. For these patients, an index date was randomly selected from the potential index dates that met the following criteria: 1) patients had at least one PsO diagnosis before a potential index date (a calendar date after the first PsO diagnosis between January 2007 and March 2012), 2) patients were continuously enrolled for 12 months (study period) after a potential index date, and 3) patients were ages 18–64 years as of a potential index date. Patient health plan enrollment periods covered by health maintenance organizations (HMOs) were excluded due to incomplete information on health care costs in the database. - Moderate-to-severe PsO patients were further identified, defined as patients who received at least one nontopical systemic therapy - Moderate-to-severe PsO patients who were comorbid with PsA (PsO+PsA cohort) were further selected as those with at least 2 distinct PsA diagnoses from January 1, 2007 to the end of the **1**2-month study period. - Control patients were selected from the same OptumHealth database. Patients free of PsO (ICD-9-CM code 696.1) and PsA (ICD-9-CM code 696.0) over the entire claims history (January 1, 1999 to March 31, 2012) were identified as control candidates. The control candidates were assigned the same index dates as the selected moderate-to-severe PsO patients. Furthermore, control candidates were required to be continuously enrolled in health plans for 12 months following the index date and age 18–64 years as of the index date.   **Exclusion criteria:**   - n/a | **Results:**   - Among 1,230 matched pairs of PsO+PsA patients and controls, PsO+PsA patients had significantly more PsO/PsA-related comorbidities, with the top 3 most common in both groups being hypertension (35.8% versus 23.5%), hyperlipidemia (34.6% versus 28.5%), and diabetes mellitus (15.9% versus 10.0%). - Compared with controls, PsO+PsA patients had a higher number of distinct prescriptions filled (incidence rate ratio 2.3, P < 0.05); were more likely to have inpatient admissions (odds ratio [OR] 1.6), emergency room visits (OR 1.3), and outpatient visits (OR 62.7) (all P < 0.05); and incurred significantly higher total, pharmacy, and medical costs (adjusted annual cost differences per patient $23,160, $17,696, and $5,077, respectively; all P < 0.01). | **Common comorbidities:**   - Hypertension - Hyperlipidemia - Rheumatoid arthritis - Diabetes mellitus - Depression - Coronary heart disease - Acute myocardial infarction - Anxiety - Obesity - Cerebrovascular disease(stroke) - Occlusion and stenosis of precerebral arteries - Peripheral vascular disease - Skin cancer - Nonmelanoma - Crohn’s disease - Ulcerative colitis - Multiple sclerosis - Lymphoma - Alopecia areata - Celiac disease - Systemic sclerosis - Sjogren’s syndrome - Vitligo - Chronic urticaria - Systemic lupus erythematosus - Addison’s disease - Giant cell arteritis - Pulmonary fibrosis - Chronic glomerulonephritis   **Key Impacts:**   - Economic burden - Hospitalization/ER visits - Healthcare costs/utilization (in general)   **Unmet Needs:**   - Economic burden of therapies and increase healthcare utilization   **Access to Treatment/Care:**   - Medications and pharmacologic therapies do exist, but can be costly |
| Gronning *et al*., 2011 | **Objective:** To investigate the process of coping in people living with chronic inflammatory arthritis.  **Design:** qualitative  **Methods:**   - Semi-structured individual face-to-face interviews with 26 persons having rheumatoid arthritis, psoriatic arthritis or unspecified polyarthritis were performed. The informants were asked how they experienced to live with arthritis and how they coped with challenges due to the arthritis. | **Condition:** RA, PsA, or unspecified polyarthritis  **Country:** Norway  **Characteristics:**   - Of a total of 26 patients interviewed, 22 were female, and 6 were diagnosed with PsA.   **Inclusion criteria:**   - Patients had rheumatoid arthritis, psoriatic arthritis or unspecified polyarthritis according to the International Statistical Classification of Diseases and Related Health Problems (ICD-10) and had been treated at the Rheumatology Department at the University Hospital in Trondheim in 2007. - Patients with early inflammatory arthritis (insufficient to diagnose) were also included because they are considered to have similar self-management needs as patients with PsA and RA   **Exclusion criteria:**   - n/a | **Results:**   - The main finding was that the informants experienced the process of coping with arthritis as a dynamic, iterative, balancing process. - They balanced between different states, entitled “go on as usual,” “listen to the body”, “adjustments” and “attitude towards life.” - The informants preferred to be in a “go on as usual” state as this was seen as normal life. - However, disease fluctuations with pain, fatigue and stiffness disturbed the balance and made the informants “listen to the body”, a state where they became aware of how the disease affected them, followed by the “adjustment” state. - Adjustments were composed of different efforts to ease the arthritis influence and for regaining balance. - The “attitude towards life” influenced the overall process of coping. A redefined view of what the informants considered to be normal life thus happened through longer periods of imbalance. | **Key Symptoms:**   - Pain - Balance - Stiffness - Fatigue   **Key Impacts:**   - Coping - Disruption to “normal life” |
| Lebwohl *et al.,* 2014 | **Objective:** To further the understanding of the unmet needs of psoriasis and PsA patients.  **Design:** multinational, cross-sectional survey  **Methods:**   - A large, multinational, population-based survey of psoriasis and/or PsA patients in North America and Europe. - Patients were selected by list-assisted random digit dialing and did not have to currently be under the care of a health care provider, a patient organization member, or receiving treatment; 139,948 households were screened and 3426 patients completed the survey. | **Condition:** PsO and PsA  **Country:** USA, Canada, France, Germany, Italy, Spain, UK  **Characteristics:**   - Household prevalence of psoriasis and/or PsA ranged from 2.9% in France to 6.5% in Canada; extrapolated population prevalence ranged from 1.4% in Spain to 3.3% in Canada. - Of the 3426 patients surveyed, 79% indicated a current diagnosis of psoriasis alone and 21% had PsA with or without psoriasis. - Mean age was 54.8 years, and majority of patients were female (59%) and overweight or obese (64%).   **Inclusion criteria:**   - Household members >18 years of age were asked to participate in the full survey if they had ever been diagnosed with psoriasis and/or PsA by an HCP   **Exclusion criteria:**   - n/a | **Results:**   - The prevalence of psoriasis/PsA ranged from 1.4% to 3.3%; 79% had psoriasis alone and 21% had PsA. - In patients with PsA, an average of 5 years elapsed between sign/symptom onset and diagnosis. - When rating disease severity at its worst, 27% (psoriasis) and 53% (PsA ± psoriasis) of patients rated it as severe. - Psoriasis patients indicated that their most bothersome signs or symptoms were itching (43%), scales (23%), and flaking (20%). - PsA patients with > 4 affected joints answered ‘‘much difficulty’’ or ‘‘unable to do’’ for bending down to pick up clothing from the floor (26%), walking outdoors on flat ground (18%), dressing themselves (15%), getting in and out of bed or the car (15%), washing and drying their body (12%), turning faucets on and off (8%), and lifting a full cup or glass to their mouth (7%). - Of psoriasis patients, 45% had not seen a physician in a year; [80% of psoriasis patients with $4 palms body surface area and 59% of PsA patients were receiving no treatment or topical treatment only. - Of patients who had received oral or biologic therapy, 57% and 45%, respectively, discontinued therapy, most often for safety/tolerability reasons and a lack/loss of efficacy. | **Key Symptoms:**   - Itching - Scales - Redness/irritation - Flaking - Lesions - Joint pain   **Key Impacts:**   - Limitations to mobility - ADLs - IADLs - Cost of treatment   **Common Comorbidities:**   - Arthritis - Cancer - Crohn’s disease - Depression - Diabetes - Heart disease - Hypertension - Liver disease - Ulcerative colitis - Uveitis   **Unmet Needs:**   - Delay to diagnosis - Managing limitations to mobility   **Access to Treatment/Care:**   - In patients with PsA, an average of 5 years elapsed between sign/symptom onset and diagnosis - 59% of PsA patients were receiving no treatment or topical treatment only. - Of patients who had received oral or biologic therapy, 57% and 45%, respectively, discontinued therapy, most often for safety/tolerability reasons and a lack/loss of efficacy. |
| Lebwohl *et al.,* 2016 | **Objective:** To report MAPP results from US physicians and patients.  **Design:** cross-sectional survey  **Methods:**   - Adults were contacted by household telephone, using random digit dialing, and asked to participate if they had ever been diagnosed with psoriasis or PsA. - Physicians were identified through national databases and contacted through random sampling methods. | **Condition:** PsO and PsA  **Country:** USA  **Characteristics:**   - In the USA, 1005 patients, 101 dermatologists, and 100 rheumatologists were surveyed. - PsA had been diagnosed in 270 patients (26.9 %). - Mean age of surveyed patients was 57 years, and 61.3 % were female   **Inclusion criteria:**   - Household members >18 years of age were asked to participate in the full survey if they had ever been diagnosed with psoriasis and/or PsA by an HCP   **Exclusion criteria:**   - n/a | **Results:**   - Of those with psoriasis alone, fewer than 60 % (versus 85.6 % of PsA patients) had seen a healthcare provider within 12 months. - Joint pain was reported by 51.8 %of psoriasis patients without a diagnosis of PsA, and 37.6 % of dermatologists cited their greatest challenge in managing PsA patients as being differentiating PsA from other arthritic diseases. - Itching was reported by 36 % of psoriasis patients versus 12 % of dermatologists as the most important factor contributing to disease severity. - Patients reported lower rates of current treatment than did dermatologists and rheumatologists. Conventional oral and biologic therapies were used by 24.9 and 17.7 % of patients, respectively. - Among patients who had received injectable biologics, treatment dissatisfaction was related to long-term safety/tolerability, injection-related anxiety/fear, and cost. | **Key Symptoms:**   - Pain - Joint swelling - Itching - Scaling - Flaking - Lesions   **Key Impacts:**   - Medical costs   **Common Comorbidities:**   - Arthritis - Cancer - Crohn’s disease - Depression - Diabetes - Heart disease - Hypertension - Liver disease - Ulcerative colitis - Uveitis   **Unmet Needs:**   - Screening, assessing, diagnosing, and treating PsO patients who have symptoms of PsA - PsA is undertreated in patient with moderate to severe disease - Managing pain - Managing limitations to mobility - Treatment satisfaction/compliance (e.g., fear of injectables)   **Access to Treatment/Care:**   - Both psoriasis and psoriatic arthritis remain undertreated in patients with moderate to severe disease. - Widespread dissatisfaction with current treatment options is reflected in patients not seeing a physician or not initiating or continuing therapies because of concerns with long-term safety, administration challenges, and cost. - Differences in perceptions between patients and physicians regarding disease severity and its impact on treatment selection, and highlight the need for ongoing communication between patients and physicians for better understanding of perceptions of disease severity, as well as treatment options and goals. - Patients show concern regarding adverse effects of therapies |
| McDonough *et al.,* 2014 | **Objective:** (1) To determine the prevalence of depression and anxiety in patients with psoriatic arthritis (PsA) and to identify associated demographic and disease-related factors. (2) To determine whether there is a difference in the prevalence of depression and anxiety between patients with PsA and those with psoriasis without PsA (PsC).  **Design:** cross-sectional  **Methods:**   - Consecutive patients attending PsA and dermatology clinics were assessed for depression and anxiety using the Hospital Anxiety and Depression Scale. - Patients underwent a clinical assessment according to a standard protocol and completed questionnaires assessing their health and quality of life. - T tests, ANOVA, and univariate and multivariate models were used to compare depression and anxiety prevalence between patient cohorts and to determine factors associated with depression and anxiety. | **Condition:** PsA and PsC  **Country:** Canada  **Characteristics:**   - Assessed 306 patients with PsA and 135 with PsC. - The age of patients with PsA and those with PsC was similar (53.8 and 52.4 yrs, respectively). The mean age at diagnosis for psoriasis was significantly younger in the PsA group (27.5 yrs vs 30.9 yrs for patients with PsC), and the duration of psoriasis was longer (mean duration 26.4 yrs vs 21.6 yrs). - There were significantly more men in the PsA group (61.4% vs 48% with PsC) and they were more likely to be unemployed.   **Inclusion criteria:**   - PsA satisfied the Classification Criteria for Psoriatic Arthritis (CASPAR). - Patients with PsC had their psoriasis confirmed by a dermatologist and were assessed by a rheumatologist to exclude the diagnosis of PsA, and were reevaluated annually for the possible development of PsA.   **Exclusion criteria:**   - n/a | **Results:**   - Prevalence of both anxiety and depression was higher in patients with PsA (36.6% and 22.2%, respectively) compared to those with PsC (24.4% and 9.6%; p = 0.012, 0.002). - Depression and/or anxiety were associated with unemployment, female sex, and higher actively inflamed joint count as well as disability, pain, and fatigue. - In the multivariate reduced model, employment was protective for depression (OR 0.36) and a 1-unit increase on the fatigue severity scale was associated with an increased risk of depression (OR 1.5). - PsA group more likely to have taken nonsteroidal antiinflammatory drugs (NSAID), disease-modifying antirheumatic drugs (DMARD), and biologic agents than patients with PsC, but were less likely to have received ultraviolet phototherapy and topical medications for psoriasis. | **Key Symptoms:**   - Joint pain   **Key Impacts:**   - Psychological/emotional effects - Anxiety - Depression - Ability to work   **Unmet Needs:**   - Managing psychological/emotional effects - Under-recognized/undertreated depression and anxiety   **Access to Treatment/Care:**   - PsA group more likely to have taken nonsteroidal antiinflammatory drugs (NSAID), disease-modifying antirheumatic drugs (DMARD), and biologic agents than patients with PsC, but were less likely to have received ultraviolet phototherapy and topical medications for psoriasis. |
| Michelsen *et al.,* 2015 | **Objective:** To compare disease burden in rheumatoid arthritis (RA), psoriatic arthritis (PsA) and axial spondyloarthritis (ax-SpA).  **Design:** cross-sectional  **Timeframe:** 2013  **Methods:**   - Patient-reported health status, demographic variables, medications, and composite scores of disease activity were assessed. - The main analyses were performed using General Linear Models adjusted for age, sex and multiple comparisons. - Correlation analyses were performed using Spearman’s rho. | **Condition:** RA, PsA, and AxSpA  **Country:** Norway  **Characteristics:**   - 66.8% (682/1021) of the RA patients were rheumatoid factor (RF) positive, 69.4% (697/1004) anti-citrullinated peptide antibodies (ACPA) positive and 75.3% (747/992) positive to RF, ACPA or both. The PsA patients (365) all fulfilled the ClASsification for Psoriatic ARthritis (CASPAR) criteria [21]. PsA patients with axial inflammation were counted to the ax-SpA group. Patients with AS (266), PsA with axial inflammatory affection (22) or nonradiographic ax-SpA (45) were included in the ax-SpA group. They all (333) fulfilled the ASAS classification criteria for ax-SpA [8]. 85.6% (n = 250/292) of the ax-SpA and 27.1% (n = 45/ 166) of the PsA patients were HLA-B27 positive. - 1093 RA, 365 PsA and 333 ax-SpA patients - The ax-SpA patients (48±12.9 years) were significantly (p<0.001)   younger than the PsA (55±12.4 years) and the RA patients (63±13.8 years).   - Although the RA patients were older than the ax-SpA patients, the two groups did not differ in disease duration (RA: 12.4±10.6 years, ax-SpA: 13.0±11.8 years, p = 0.777). The PsA patients had significantly (p<0.001) shorter disease duration (9.9 ± 8.2) than the RA and the ax-SpA patients. - The RA patients had significantly (p<0.001) less education (11.4±3.6 years) than the PsA (12.4±3.6 years) and the ax-SpA patients (12.8±3.5 years). - The RA patients were predominantly female (68.5%), the ax-SpA patients predominantly male (66.7%), whereas the gender distribution in the PsA group was equally balanced with 49.3% female.   **Inclusion criteria:**   - All the RA (1093), PsA (365) and ax-SpA (333) patients who visited the out-patient clinic of the Hospital of Southern Norway Trust during the year 2013 were included. - The RA patients all had a RA diagnosis verified by the treating rheumatologist, the PsA patients all fulfilled the ClASsification for Psoriatic ARthritis (CASPAR) criteria and the ax-SpA patients all fulfilled the Assessment of SpondyloArthritis international Society (ASAS) classification criteria for ax-SpA.   **Exclusion criteria:**   - n/a | **Results:**   - The reported pain, joint pain, patient’s global assessment and fatigue were similar in PsA and ax-SpA, but significantly lower in RA. - The 28-joint Disease Activity Score (DAS28) (0.3±0.1, p = 0.003), Clinical Disease Activity Index (CDAI) (1.0±0.4, p = 0.028) and Routine Assessment of Patient Index Data 3 (RAPID3) (0.4±0.1, p = 0.004) were all significantly higher in PsA vs. RA. - RAPID3 showed moderate to high correlation with DAS28 (rho = 0.521, p<0.001) and CDAI (rho = 0.768, p<0.001) in RA and PsA, and with Bath Ankylosing Spondylitis Disease Activity Index (BASDAI) (rho = 0.902, p<0.001) and Bath Ankylosing Spondylitis Functional Index (BASFI) (0.865, p<0.001) in ax-SpA and PsA. - The current use of bDMARDs was significantly more frequent (p = 0.001) in the ax-SpA (45.3%), than in the PsA (33.4%) and the RA group (34.5%). Former use of bDMARDs was also more frequent (p = 0.002) in the ax-SpA (53.8%) than in the PsA (41.4%) and the RA group (44.4%). - The current use of TNF inhibitors was 45.0% in the ax-SpA, 31.8% in the PsA and 21.0% in the RA group. Previous and current use of csDMARDs was more frequent in the RA group compared to the PsA group (previous use: 92.1% versus 84.4%, p<0.001, current use: 61.1% versus 52.9%, p = 0.003). | **Key Symptoms:**   - Pain - Morning stiffness - Joint issues - Fatigue   **Key Impacts:**   - Ability to work   **Access to Treatment/Care:**   - The current use of TNF inhibitors was 31.8% in the PsA group, and current use of bDMARDs was 33.4%. - Former use of bDMARDs was 41.4% in PsA group. |
| Moverley *et al.,* 2015 | **Objective:** To explore patients’ experience of flare in PsA  **Design:** Qualitative interview study  **Methods:**   - N=18, in-person, Semi-structured interviews in an informal clinical setting lasting between 25 min to 2hrs - Clinician performed interviews, but without an on-going doctor-patient relationship - Patients recruited from a Rheumatology clinic and those included were specifically selected because they recently or previously experienced significant peripheral disease flare - Patients approached by phone or at a visit and those interested were scheduled for a later interview - IRB approval and Informed consent was received - Interviews were audio-recorded and transcribed - A thematic analysis was performed using NVivo | **Condition:** PsA  **Country:** England  **Characteristics:**   - Approx. 1/3 (n=5) of patients currently experienced a flare - Mean age of 44 yrs - Avg. disease duration: 4.5 years - Males: n=9 - Experiencing significant skin burden: n=4 - Experiencing significant axial burden: n=4   **Inclusion criteria:**   - All patients fulfilled classification criteria for PsA (CASPAR) - Experienced a recent or previous significant peripheral disease flare - Included some patients who experienced significant skin and/or axial burden   **Exclusion criteria:**  n/a | **Results:**   - 9 main overarching themes emerged (physical symptoms, fatigue, social withdrawal, psychological symptoms, loss of normal function, triggers, management of pre-flare, management of flare and timing of flare) - Physical symptoms were a significant burden during flares - Psoriasis will also flare during a flare - Fatigue was a significant burden; caused many of the psychological impacts - Patients identified flare triggers (e.g., damp weather, doing more physical work) - Duration of flare was variable - Patients could identify a bad day coming on (e.g., up and down a lot during the night) - Longer disease experience led to greater disease awareness and understanding of triggers and warning signs - Patients managed flares through self-medicating, self-help, seeking medical attention, avoiding things, trying to understand the disease, or just “getting on with it.” - Patients learn to self-manage and avert full flare - Medical help sought when all else fails - Physical symptoms occur in combination - Data saturation reached by patient 11 - One patient described a permanent state of flare | **Key Symptoms:**   - Joint swelling - Pain - Heat - Stiffness - Fatigue   **Key Impacts:**   - Loss of normal functioning - Social withdrawal - Lack of desire for physical contact - Grooming - Loss of independence/increased dependence on others - Loss of movement - Loss of hobbies - Psychological impacts (frustration, depression, embarrassment, fear) - Relationships with family/spouse |
| Pentek *et al.*, 2014 | **Objective:** To provide an overview of biological treatment in six Central and Eastern European (CEE) countries: Bulgaria, Czech Republic, Hungary, Poland, Romania and Slovakia  **Design:** Literature Review  **Methods:**   - Literature summarized according to epidemiology, disease burden and use of biological agents in 3 rheumatologic conditions - No additional methodology described | **Conditions:** Rheumatoid arthritis (RA), Ankylosing Spondylitis (AS), Psoriatic Arthritis (PsA)  **Countries:** Bulgaria, Czech Republic, Hungary, Poland, Romania and Slovakia  **Characteristics:** N/A  **Inclusion Criteria:** N/A  **Exclusion Criteria:** N/A | **Results:**   - Estimated prevalence of AS in Europe is 23.8 per 10,000 with an estimated number of 1.3-1.5 million cases - Prevalence of PsA varies by country and region; Prevalence of psoriasis in the general pop. Is approx. 2-3%, with about a third having arthritis - Lack of epi data on CEE countries - AS can have important socioeconomic burden on individuals and society - AS-related sick days estimated between 6.5-18 days per patient/per year - 15-20%of AS patients require help from relatives/others - Loss of work productivity increases costs to patient and society - Most important predictor in AS for high costs is functional disability - In CR, two studies showed mean annual costs per AS patient was EU4,782 (Beda I) and EU5,806 (Beda II), with direct costs estimated at EU1,812 (Beda I) and EU2,588 (Beda II). Largest expenses were spa procedures (45.3%, Beda I) and biologic treatments (52.8%, Beda II). - Phyisotherapy is recommended for treatment of AS in Hungary. - NHIFA spent EU17.9 million on physiotherapy and biological treatments across diagnoses in 2011. NHIFA expenditure on biological drugs for AS and PsA patients was EU15.5 million in 2010 in Hungary. - Few large scale, prospective, observational studies conducted in PsA and only a few collected data on economic outcomes or patient preferences based on QoL scores. - In Germany, mean annual per patient direct cost in PsA was EU3,156; indirect cost between EU2,414 to EU7,919. - PsA disease activity and impairment of physical function main cost drivers - Total PsA related costs in Hungary per patient/per year were EU5,574 - EMA has approved 5 biological treatments for AS (adalimumab, certolizumab, pegol, etanercept, golimumab, and infliximab) and 6 for PsA (same as those for AS plus ustekinumab) - Biological treatment reimbursed by health insurance in CEE countries and therapy is available for patients under 100% coverage, but not all biological drugs are financed in all countries - Fair geographic accessibility to treatment centers - Access to biologic treatment can vary widely by country - RA patients are generally in poorer state of health than Western European countries, with one explanatory factor being the slower and more limited uptake of biological treatments - Despite centralized European guidelines and drug registration, there are significant variances in financing practices across Europe. | **Key Impacts:**   - Loss of work days/productivity - Dependence on others - Increased costs to individual patient and society   **Access to Treatment/Care:**   - Not all EMA approved biological treatments are financed by insurance in all CEE countries - Fair geographic accessibility to treatment centers - Access to biological treatment can vary widely by country - Generally slower and more limited uptake of biological treatment in CEE compared to Western Europe |

# References

1. Armstrong AW, Schupp C, Wu K, Bebo B. Quality of life and work productivity impairment among psoriasis patients: findings from the National Psoriasis Foundation survey data 2003-2011. PLoS One. 2012;7(12):e52935.
2. Chisholm A, Pearce CJ, Chinoy H, Warren RB, Bundy C. Distress, misperceptions, poor coping and suicidal ideation in psoriatic arthritis: a qualitative study. Rheumatology (Oxford). 2016 Jun;55(6):1047-52.
3. Edson-Heredia E, Zhu B, Guo J, Maeda-Chubachi T, Lebwohl M. Disease burden and quality of life in psoriasis patients with and without comorbid psoriatic arthritis: results from National Psoriasis Foundation panel surveys. Cutis. 2015 Mar;95(3):173-8.
4. Feldman SR, Zhao Y, Shi L, Tran MH, Lu J. Economic and comorbidity burden among moderate-to-severe psoriasis patients with comorbid psoriatic arthritis. Arthritis Care Res (Hoboken). 2015 May;67(5):708-17.
5. Grønning K, Lomundal B, Koksvik HS, Steinsbekk A. Coping with arthritis is experienced as a dynamic balancing process. A qualitative study. Clin Rheumatol. 2011 Nov;30(11):1425-32.
6. Lebwohl MG, Kavanaugh A, Armstrong AW, Van Voorhees AS. US Perspectives in the Management of Psoriasis and Psoriatic Arthritis: Patient and Physician Results from the Population-Based Multinational Assessment of Psoriasis and Psoriatic Arthritis (MAPP) Survey. Am J Clin Dermatol. 2016 Feb;17(1):87-97.
7. Lebwohl MG, Bachelez H, Barker J, Girolomoni G, Kavanaugh A, Langley RG, et al. Patient perspectives in the management of psoriasis: results from the population-based Multinational Assessment of Psoriasis and Psoriatic Arthritis Survey. J Am Acad Dermatol. 2014 May;70(5):871-81.e1-30.
8. McDonough E, Ayearst R, Eder L, Chandran V, Rosen CF, Thavaneswaran A, et al. Depression and anxiety in psoriatic disease: prevalence and associated factors. J Rheumatol. 2014 May;41(5):887-96.
9. Michelsen B, Fiane R, Diamantopoulos AP, Soldal DM, Hansen IJ, Sokka T, et al. A comparison of disease burden in rheumatoid arthritis, psoriatic arthritis and axial spondyloarthritis. PLoS One. 2015 Apr 8;10(4):e0123582.
10. Moverley AR, Vinall-Collier KA, Helliwell PS. It’s not just the joints, it’s the whole thing: qualitative analysis of patients’ experience of flare in psoriatic arthritis. Rheumatology (Oxford). 2015 Aug;54(8):1448-53.
11. Péntek M, Poór G, Wiland P, Olejárová M, Brzosko M, Codreanu C, et al. Biological therapy in inflammatory rheumatic diseases: issues in Central and Eastern European countries. Eur J Health Econ. 2014 May;15 Suppl 1:S35-43.
