# Supplementary material for: Patient perspectives on the pathway to psoriatic arthritis diagnosis: results from a web-based survey of patients in the United States
Source: BMC Rheumatol. 2020 Jan 10;4:2. doi: 10.1186/s41927-019-0102-7 (PMC6953285; doi:10.1186/s41927-019-0102-7)
Supplement: Supplementary file 3 — Additional file 3: Table S3. Concept elicitation from interviews with clinical experts and adults with PsA. [file 41927_2019_102_MOESM3_ESM.docx]

**Supplementary Table 3.** Concept elicitation from interviews with clinical experts and adults with PsA

|  | **Characteristics** |
| --- | --- |
| **Symptoms  (N = 5)** | Back pain (n = 5) |
|  | Pelvic pain (n = 5) |
|  | Awkward gait (n = 5) |
|  | Walking with a limp (n = 5) |
|  | Lost ability to be athletic (n = 5) |
|  | Foot problem (n = 5) |
|  | Knees hurt (n = 5) |
|  | Difficulty walking up the stairs (n = 5) |
|  | Stiffness in back (n = 5) |
|  | Respiratory issues (n = 5) |
|  | Nail pitting (n = 5) |
|  | Trouble walking (n = 5) |
|  | Inflammation in knee (n = 5) |
|  | Swelling fingers and toes (n = 5) |
|  | Fatigue (n = 5) |
|  | Skin issues (n = 5) |
|  | Swollen joints (n = 5) |
| **Impacts  (N = 5)** | Unable to do many activities |
|  | Adjusted by managing energy and being selective about activities (n = 1) |
|  | Adjusted by using ergonomic tools |
|  | Limits life (n = 1) |
|  | Having a plan for different types of days with regard to symptoms |
|  | Getting people to take you seriously about pain |
|  | Managing pain |
|  | Providers not communicating |
|  | Dropping out of able-bodied life |
|  | Gap between desire and ability |
|  | Stressful knowing what may happen in the future |
| **Current Treatment  (N = 5)** | **Biologics** |
|  | Enbrel |
|  | Cosentyx (n = 1) |
|  | Remicade (n = 2) |
|  | Humira (n = 1) |
|  | **NSAIDS** |
|  | Tylenol, Motrin, or Ibuprofen (n = 2) |
|  | Mobic |
|  | Naproxen/Prescription Aleve (n = 1) |
|  | Lidocaine |
|  | Muscle Relaxers |
|  | Ibuprofen and Nabumetone |
|  | Hydrocodone/Acetaminophen |
|  | Tylenol Arthritis (n = 1) |
|  | Naprosyn (n = 1) |
|  | Advil (n = 1) |
|  | Voltran gel (n = 1) |
|  | **Other** |
|  | Supplements (n = 1) |
|  | Compression Socks |
|  | Leucovorin (n = 1) |
|  | Acupuncture (n = 1) |
|  | Xanax (n = 1) |
|  | **Corticosteroids** |
|  | Methotrexate (n = 2) |
|  | Prednisone (n = 3) |
|  | **DMARDS** |
|  | Leflunomide (n = 1) |
| **Treatment Likes and Dislikes  (N = 5)** | **Treatment Likes** |
|  | Only have to take every two weeks (n = 2) |
|  | Inexpensive (n = 2) |
|  | Remicade helps with skin issues (n = 1) |
|  | Met nice people while getting infusions (n = 1) |
|  | Use it when I need it (n = 1) |
|  | Temporary help that works well (n = 1) |
|  | No side effects (n = 1) |
|  | Freedom of not having to think about it (n = 1) |
|  | Effectiveness (n = 1) |
|  | Easy to take (n = 1) |
|  | Don't have to inject (n = 1) |
|  | Don’t have to worry about refrigeration (n = 1) |
|  | Works well |
|  | Only once a month |
|  | Likes going to get infusions |
|  | Helps the patient |
|  | Doesn't make patient sick |
|  | **Treatment Dislikes** |
|  | Long term side effects (n = 1) |
|  | Needle (n = 1) |
|  | Time it takes (n = 1) |
|  | Methotrexate upsets stomach (n = 1) |
|  | Doesn't put in remission (n = 1) |
|  | Makes patient hungry (n = 1) |
|  | Can’t sleep (n = 1) |
|  | Cost (n = 1) |
|  | Injectable |
|  | Preauthorization process for insurance (n = 1) |
|  | Nothing (n = 1) |
|  | Shots are painful |
|  | Meds don’t always work |
|  | Have a new medication every 6 months |
|  | Having to take medication to treat side effects of medication |
|  | Feel like passing out |
|  | Spinning feeling |
|  | Hard to live with |
|  | Try to eat all day to prevent it |
|  | Fatigue |
|  | Naeusea |
|  | Reminder that patient isnt healthy |
|  | Frequency of pharmacy visits |
|  | Don’t like taking medications |
|  | Fear of side effects |
|  | Time required for infusions |
| **Ideal Treatment Characteristics (N = 5)** | Infrequent Infusions (n = 4) |
|  | Infrequent injections |
|  | Keeps you at one level all the time |
|  | Can do injections anywhere |
|  | No pills |
|  | Don't have to refrigerate injections |
|  | Don't have to warm up injections |
|  | Oral medication |
|  | Doesn't affect internal organs |
|  | Doesn't kill patient |
|  | Take once or twice a day |
|  | No nausea or dizziness |
|  | Helps patient |
|  | Know more about side effects (n = 1) |
|  | Efficacy to last until next dose (n = 1) |
|  | Injections don't hurt (n = 1) |
|  | Injections at home (n = 1) |
|  | More research on self-help (n = 1) |
|  | Oral medication (n = 1) |
|  | No side effects (n = 1) |
|  | Reduce/eliminate breakthrough tendonitis/bursitis (n = 1) |
| **Burden in Taking Current Medication  (N = 5)** | No burden (n = 4) |
|  | Remembering to take meds |
|  | Time required for infusion (n = 1) |
|  | Timing when taking pills with breakfast |
|  | Anxious about having to inject self |
|  | Having to eat breakfast so that I can take pills |
|  | Hate taking pills |
|  | Partner has to inject me |
|  | Takes time for medication to warm up |
|  | Always there |
|  | Takes time to rub medicaiton on |
|  | Do not like taking medications |
|  | Upset when looking at medications |
|  | Fear of potential side effects |
|  | Makes me sick (n = 1) |
|  | Limits alcohol consumption (n = 1) |
|  | Cannot stand feeling of syringe |
| **Access to Care (N = 5)** | **Overall** |
|  | Good/no problems (n = 2) |
|  | Gotten better since I have moved to bigger city |
|  | OK for most part except for cost |
|  | Very barriered |
|  | Constantly fighting for access to treatment |
|  | Poster child for easy access to medical care |
|  | Negative (n = 1) |
|  | Not pleased only have choice of one doctor (n = 1) |
|  | Pretty lucky--few barriers (n = 1) |
|  | **Distance to doctor or pharmacy** |
|  | No issues (n = 4) |
|  | Prescriptions delivered and doctors are close (n = 2) |
|  | Doctor 2 hours away (n = 1) |
|  | **Inability to see specialist** |
|  | No issues (n = 4) |
|  | 6 months to get an appt (n = 1) |
|  | Easy to get in to see a doctor (n = 1) |
|  | **Cost/insurance** |
|  | No issues (n = 3) |
|  | Issues with getting authorization from insurance (n = 2) |
|  | Drugs are too expensive (n = 1) |
|  | Have to pay a large amount every few months (n = 1) |
|  | Have to get financial assistance from pharmaceutical companies (n = 1) |
|  | Medicaid does not pay for some drugs (n = 1) |
|  | Affects seeing specialists |
|  | Cannot afford certain treatments right now |
|  | **Transportation** |
|  | No issues (n = 5) |
|  | Used to have issues in the past, but not now (n = 1) |
|  | Unable to drive |
|  | Laborious to get to appts |
|  | Not mentioned |
| **Unmet Needs (N = 5)** | **Meaning of supportive services** |
|  | Support groups (n = 2) |
|  | Financial assistance (n = 2) |
|  | Going on disability |
|  | Therapist |
|  | Nutritionist |
|  | Helping me (n = 1) |
|  | Physical therapy (n = 1) |
|  | Education classes (n = 1) |
|  | Social services (n = 1) |
|  | **Patient counseling** |
|  | Yes, an unmet need (n = 1) |
|  | Geared more towards young adults |
|  | Wish I knew how bad it was going to be |
|  | No, not an unmet need (n = 4) |
|  | Had to go out on own and explore |
|  | Would have probably liked it when first diagnosed though |
|  | **Disease education** |
|  | Yes, an unmet need (n = 1) |
|  | Difficulty knowing if what's online is accurate |
|  | Steps for day-to-day living and happiness |
|  | How to have hobbies |
|  | How to go on vacation |
|  | How to still enjoy life |
|  | Would have been helpful at beginning (n = 1) |
|  | Wanted to know more about fatigue |
|  | No, not an unmet need (n = 3) |
|  | **Support groups** |
|  | Yes, an unmet need (n = 2) |
|  | Online |
|  | In person (n = 1) |
|  | Geographically close to me (n = 1) |
|  | I invented it |
|  | In person group for minorities |
|  | No, not an unmet need (n = 3) |
|  | Started a group |
|  | **Other unmet needs** |
|  | Maid |
|  | Handicap sticker |
|  | More resources for younger people |
|  | Information on how to do treatments |
|  | Information for how to do things when brain fog hits |
|  | Health/life coach |
|  | Nutritionist |
|  | Exercise programs |
|  | Alternative ways to modify things (n = 1) |
|  | None (n = 1) |
| **Current Symptoms  (N = 5)** | **Spontaneous** |
|  | Fatigue (n = 3) |
|  | Painful/swollen joints (n = 5) |
|  | General pain |
|  | Psoriasis (n = 3) |
|  | Pain in lower back/hip |
|  | Uveitis/iritis (n = 1) |
|  | Sausage-like fingers/toes (n = 2) |
|  | Plantar fasciitis (n = 1) |
|  | Achilles tendonitis (n = 1) |
|  | Joint stiffness (n = 1) |
|  | **Probed** |
|  | Uveitis/iritis (n = 4) |
|  | Joint stiffness (n = 4) |
|  | Psoriasis (n = 3) |
|  | Achilles tendonitis (n = 2) |
|  | Fatigue (n = 1) |
|  | Sausage-like fingers/toes (n = 2) |
|  | Pain in lower back/hip |
|  | Rash (n = 1) |
|  | Back pain (n = 1) |
|  | Hip pain (n = 1) |
|  | Nausea (n = 1) |
|  | Swollen wrist (n = 1) |
|  | Plantar fascitis |
|  | Painful/swollen joints |
