# Supplementary material for: Patient perspectives on the pathway to psoriatic arthritis diagnosis: results from a web-based survey of patients in the United States
Source: BMC Rheumatol. 2020 Jan 10;4:2. doi: 10.1186/s41927-019-0102-7 (PMC6953285; doi:10.1186/s41927-019-0102-7)
Supplement: Supplementary file 4 — Additional file 4. Supplemental Appendix -- Survey. [file 41927_2019_102_MOESM4_ESM.docx]

Thank you for your interest in participating in this study. First, we need to ask you a few questions in order to determine if you are eligible to participate.

Please click on a response option to select your answer.

1. Have you been diagnosed by a physician with any of the following conditions? Please select all that apply.

**[Respondent must select psoriatic arthritis]**

**[RANDOMIZE]**

- Psoriatic arthritis **[Screen out if not selected] [ANCHOR]**
- Ankylosing spondylitis
- Alzheimer’s disease **[screen out if selected]**
- Dementia **[screen out if selected]**
- Diabetes
- Attention Deficit/Hyperactivity Disorder (ADHD)
- Colon cancer
- Asthma
- Seasonal allergies
- Heartburn
- Gastroesophageal Reflux Disease (GERD)
- Hypertension (high blood pressure)
- None of the above **[ANCHOR]** **[screen out if selected]**

**[Next screen]**

1. What is your age?

______ **[show dropdown list, 0-99]** **[Screen out if under 18]**

**[Next screen]**

1. Who do you **currently** see to treat your psoriatic arthritis? Please select all that apply.

**[SCREEN OUT if RHEUMATOLOGIST or DERMATOLOGIST is not selected]**

- Dermatologist
- Rheumatologist
- General practitioner/family doctor
- Orthopedist
- Podiatrist
- Sports medicine specialist
- Chiropractor
- Psychologist
- Other
- Don’t know **[screen out]**

**[Next screen]**

1. What is your current country of residence?

**[Add drop down menu with years]**

**[SCREEN OUT if US is not selected]**

**[Next screen]**

1. Would you be willing to complete an online survey that lasts about 20 minutes?
   - Yes
   - No **[screen out if selected]**

**[Next screen]**

**[IF RESPONDENT IS ELIGIBLE, PROCEED WITH THE FOLLOWING RECRUITMENT MESSAGE]**

Based upon the answers you provided, you are eligible to participate in an online survey about your experiences with treating and managing psoriatic arthritis.

Length of survey: About 20 minutes

Payment: **[Insert relevant amount]**

**Survey is open only until we have reached the number of participants that we would like to complete the survey. Don’t miss this opportunity!**

The survey is designed to be completed in one session. However, if you need to leave part-way through the survey, just click the link provided in the original email invitation, and you will be returned to where you left off. You will be paid after completing and submitting the survey, so please make sure that you complete the survey as quickly as possible to avoid being closed out of this opportunity.

If you are ready to begin now, click the arrow button below to read more about the study and provide consent to participate.

**[Include “back” and “next” buttons at the bottom of each screen]**

**[Next screen]**

**WELCOME**

We appreciate you taking the time to complete this survey. Your opinion is very important to us.

The purpose of this survey is to learn about your experiences with psoriatic arthritis, treatment and your communications with your healthcare providers.

The next screen will tell you more about the study, including any risks and benefits of participating. Please read this page carefully, and, if you still would like to participate, please select the option at the bottom of the next screen indicating that you agree to take this survey.

**[Next screen]**

**[Insert ICD]**

# AGREEMENT TO PARTICIPATE

This consent document contains important information to help you decide if you want to be in this study. If you have any questions that are not answered in this consent document, please contact the Principal Investigator.

I have read and understand the above information. I hereby voluntarily consent and offer to take part in this study and give permission to use the information I provide to take part in the study.

Check one box:

**Print a copy of this consent to keep for your records.**

- I have read the above statement, and I agree to participate in this study.
- I have read the above statement, and I do not agree to participate in this study. **[If selected, show: You’ve indicated that you do not agree to participate in this study. Is that correct? Yes/No. If YES, END SURVEY]**

**[Next screen]**

**INTRODUCTION**

Thank you for agreeing to participate in this survey. Before you begin, please note that the survey will take about 20 minutes to complete. If you need to leave the survey part-way through, you can re-enter the survey at the point where you left off using the survey link.

If you have any questions about the study or the survey, or if you need technical assistance, please call:

**[Insert number]** (Monday-Friday, **[Insert available times]**).

We recommend writing this number down or printing this page before you begin the survey.

Your responses will be kept strictly confidential, and we ask you to be as honest as possible when answering the questions. We are very interested in your opinions. There are no right or wrong answers. The survey will begin on the next screen.

**[Next screen]**

**Section I: Questions about You**

First, we’d like to ask a few questions to learn more about you.

**[Next screen]**

1. What is your sex?
   - Male
   - Female
   - Intersex

**[Next screen]**

1. Are you Hispanic or Latino?
   - Yes
   - No
2. Which of the following best describes your racial background?

**[All options are exclusive choice]**

- White (Origins in Europe, Middle East, North Africa)
- Black/African American (Origins in Black racial groups of Africa)
- Asian (Origins in Far East, Southeast Asia, Indian subcontinent)
- American Indian/Alaska Native (Origins in original peoples of North America who maintain tribal affiliations)
- Native Hawaiian/Other Pacific Islander (Origins in Hawaii, Guam, Samoa or other Pacific Islands)
- Multiracial
- Prefer not to answer

**[Next screen]**

1. What is the highest level of education you have completed?

**[All options are exclusive choice]**

- Less than high school
- High School/GED
- Some college (no degree)
- Associate’s degree
- Trade school/Certificate program
- Undergraduate degree (Bachelor’s)
- Post-graduate degree (Master’s, Doctoral)

**[Next screen]**

1. How would you describe your current employment status? Please select the answers that best apply.

**[Only allow up to 2 responses]**

- Employed, full-time
- Employed, part-time
- Self-employed
- Stay-at-home parent/homemaker
- Unemployed
- Retired
- Student
- Disabled
- Other

**[Next screen]**

1. Please select the option below which best describes your current relationship status:

**[All options are exclusive choice]**

- Married
- Separated
- Divorced
- Widowed
- Partnered
- Single
- Other

**[Next screen]**

1. Which of the following best describes your current annual household income?

**[All options are exclusive choice]**

- Less than $25,000
- $25,000 to $49,999
- $50,000 to $74,999
- $75,000 to $99,999
- $100,000 or more
- Prefer not to answer

**[Next screen]**

1. What type of health insurance do you currently have? Please select all that apply.

- Medicare
- Medicaid
- Other government insurance (for example, Tricare, CHAMPVA)
- Private insurance (purchased privately or through an employer)
- Other
- I don’t have health insurance
- I don’t know

**[Next screen]**

**Section II: You and Your Experience with Psoriatic Arthritis**

In this section of the survey, we will ask you questions about your path to receiving your diagnosis and your experience living with psoriatic arthritis. Please answer each question as best as you can.

**[Next screen]**

**Diagnosis**

1. What symptoms **first** led you to see a healthcare provider about your psoriatic arthritis? Please select all that apply.

**[RANDOMIZE]**

- Back pain
- Sciatica (radiating pain beginning in the lower back and shooting down the back of the leg)
- Foot problems (for example, plantar fasciitis, arthritis in feet, etc.)
- Joint pain
- Swollen joints
- Neck pain
- Difficulty walking
- Stiffness
- Red or inflamed eyes/uveitis (eye problems such as blurred vision, pain, or sensitivity to light)
- Fatigue/exhaustion/tiredness
- Sausage-like fingers or toes
- Tendon or ligament pain
- Skin rash/psoriasis
- Nail problems (for example, nails look infected or lift from nail bed)
- Difficulty breathing
- Chest pain
- Pelvic pain
- Reduced range of motion (difficulty bending) in your spine and/or hips.
- Reduced range of motion in the joints of your arms and legs, for example difficulty bending your elbows, fingers, and feet/toes.
- Mental health effects (for example, depression or anxiety)
- Difficulty sleeping
- Other

**[Next screen]**

1. In what year did the first symptoms of psoriatic arthritis start?

**[Add drop down menu with years]**

1. How long did these symptoms persist before you **first** sought medical care? Please provide your best estimate.

- Less than a month
- After 1-3 months
- After 4-6 months
- After 7-12 months
- After 1-2 years
- After more than 2 years

**[Next screen]**

1. What type(s) of healthcare provider(s) did you see about your symptoms during the diagnosis process? Please select all that apply.

- General practitioner/family doctor
- Pediatrician
- Rheumatologist
- Orthopedist
- Podiatrist
- Sports medicine specialist
- Dermatologist
- Chiropractor
- Psychologist/psychiatrist
- Urgent care/ER doctor
- Other
- Don’t know

**[Next screen]**

1. How long did it take from the time you **first sought medical attention** until you received a formal diagnosis for your psoriatic arthritis?

**[All options are exclusive choice; make response options a drop-down list]**

- Immediate – received diagnosis on first visit to healthcare provider
- Less than 6 months
- 6 months to 1 year
- 2 years
- 3 years
- 4 years
- 5 years
- 6 years
- 7 years
- 8 years
- 9 years
- 10 years
- Greater than 10 years

1. In what year were you **officially** **diagnosed** with psoriatic arthritis? Please provide your best estimate.

**[Answer cannot be earlier than Q10. If so, show error message: Year of diagnosis can’t be prior to year first symptoms noticed. Please check your answers]**

_____ **[insert drop-down box with years]**

**[Next screen]**

1. During the diagnosis process, were you ever misdiagnosed with a condition(s) other than psoriatic arthritis? Only think about conditions that you may have been initially diagnosed with that were inaccurate and that you do not actually have. If yes, please select only the condition(s) that was inaccurately diagnosed. Please select all that apply.

- Anxiety/depression
- Rheumatoid arthritis
- Fibromyalgia
- Bursitis
- Back problems
- Psoriatic Arthritis
- Ankylosing Spondylitis
- Osteoarthritis
- Gout or other crystal disease
- Sciatica
- Morton’s neuroma
- Orthopedic problems
- “All in my head”/psychosomatic
- Other
- I was never misdiagnosed **[Exclusive choice]**
- Don’t know

**[Next screen]**

**Current Care of Your Condition**

**[Next screen]**

1. Are you currently experiencing a flare-up of your psoriatic arthritis?
   - Yes
   - No
   - Don’t know

**[Next screen]**

1. Which of the following signs and symptoms of psoriatic arthritis do you **currently** experience? Please select all that apply.

**[RANDOMIZE]**

- Back pain
- Sciatica (radiating pain beginning in the lower back and shooting down the back of the leg)
- Foot problems (for example, plantar fasciitis, arthritis in feet, etc.)
- Joint pain
- Swollen joints
- Neck pain
- Difficulty walking
- Stiffness
- Red or inflamed eyes/uveitis (eye problems such as blurred vision, pain, or sensitivity to light)
- Fatigue/exhaustion/tiredness
- Sausage-like fingers or toes
- Tendon or ligament pain
- Skin rash/psoriasis
- Nail problems (for example, nails look infected or lift from nail bed)
- Difficulty breathing
- Chest pain
- Pelvic pain
- Reduced range of motion (difficulty bending) in your spine and/or hips.
- Reduced range of motion in the joints of your arms and legs, for example difficulty bending your elbows, fingers, and feet/toes.
- Mental health effects (for example, depression or anxiety)
- Difficulty sleeping
- Other

**[Next screen]**

1. If you had to take the palm of your hand and cover up all of the patches of psoriasis on your body today, how many palms of your hand do you think that it would take? One palm of your hand is equal to about 1% of your body surface area (BSA).If your psoriasis is only scattered small dots, try to imagine combining them together into one patch. Please remember to include your scalp and back if affected. Do not include areas in which psoriasis has faded, leaving only changes in the color of the skin. How many palms would it take to cover all of your psoriasis today?

**[All options are exclusive choice]**

- Little to no psoriasis visible (<1 palm),
- Only a few patches that could be covered by 1 to 2 palms of your hand
- Scattered patches that could be covered by between 3 and 10 palms of your hand
- Extensive psoriasis covering large areas of the body that would be more than 10 palms of your hand

1. Have you ever had any of the following health conditions? Please select all that apply.

- High blood pressure
- Coronary heart disease or heart attack
- Cerebrovascular disease or stroke
- Peripheral vascular disease
- Dyslipidemia (high or low cholesterol)
- Diabetes
- Migraines
- Sleep apnea
- Anxiety
- Depression
- Asthma
- Fibromyalgia
- Uveitis
- Inflammatory bowel disease (Crohn’s disease or ulcerative colitis)
- Irritable bowel syndrome (IBS)
- None of the above **[Exclusive Choice]**

**[Next screen]**

**Section III. Activities of Daily Life**

The following set of questions asks you about a variety of difficulties that you may or may not experience in your daily life.

**[Next screen]**

1. Over the last week, were you able to:

**[Insert following response options for each question below: Without ANY difficulty, with SOME difficulty, With MUCH difficulty, UNABLE to do]**

- - Dress yourself, including tying shoelaces and doing buttons?
  - Get in and out of bed?
  - Lift a full cup or glass to your mouth?
  - Walk outdoors on flat ground?
  - Wash and dry your entire body?
  - Bend down to pick up clothing from the floor?
  - Turn regular faucets on and off?
  - Get in and out of a car, bus, train, or airplane?
  - Walk two miles or three kilometers, if you wish?
  - Participate in recreational activities and sports as you would like, if you wish?
  - Get a good night’s sleep?
  - Deal with feelings of anxiety or being nervous?
  - Deal with feelings of depression or feeling blue?

1. How much pain have you had because of your condition OVER THE PAST WEEK? Please indicate below how severe your pain has been:

**[Insert 0-10 scale, with points at every HALF point, with 0=NO PAIN and 10=PAIN AS BAD AS IT COULD BE]**

1. Considering all the ways in which illness and health conditions may affect you at this time, please indicate below how you are doing:

**[Insert 0-10 scale, with points at every HALF point, with 0=VERY WELL and 10=VERY POORLY]**

**[Next screen]**

| **Please respond to each item by marking one box per row** | | **Excellent** | **Very Good** | **Good** | **Fair** | **Poor** |
| --- | --- | --- | --- | --- | --- | --- |
| a | In general, would you say your health is: |  |  |  |  |  |
| b | In general, would you say your quality of life is: |  |  |  |  |  |
| c | In general, how would you rate your physical health? |  |  |  |  |  |
| d | In general, how would you rate your mental health, including your mood and your ability to think? |  |  |  |  |  |
| e | In general, how would you rate your satisfaction with your social activities and relationships? |  |  |  |  |  |
| f | In general, please rate how you usually carry out your usual social activities and roles. (This includes activities at home, at work and in your community, and responsibilities as a parent, child, spouse, employee, friend, etc…) |  |  |  |  |  |
|  |  |  |  |  |  |  |
|  |  | **Completely** | **Mostly** | **Moderately** | **A little** | **Not at All** |
| g | To what extent are you able to carry out your everyday physical activities such as walking, climbing stairs, carrying groceries, or moving a chair? |  |  |  |  |  |
|  |  |  |  |  |  |  |
|  | **In the past 7 days** | **Never** | **Rarely** | **Sometimes** | **Often** | **Always** |
| h | How often have you been bothered by emotional problems such as feeling anxious, depressed or irritable? |  |  |  |  |  |
|  |  | **None** | **Mild** | **Moderate** | **Severe** | **Very Severe** |
| i | How would you rate your fatigue on average? |  |  |  |  |  |
|  | How would you rate your pain on average? | 0 1 2 3 4 5 6 7 8 9 10  No Worst pain pain  imaginable | | | | |

**[Next screen]**

**[Ask Q24 only to those who respond to being employed part-time or full-time or self-employed in Q5]**

1. Do you experience any of the following difficulties related to **work** because of your psoriatic arthritis? Please select all that apply.
   - Had to switch careers/jobs
   - Need accommodations at work
   - Need to work from home
   - Missed work
   - Frequently late to work
   - Only able to work part-time
   - Loss of productivity at work
   - Had to change work tasks
   - Difficulty doing physical tasks
   - Difficulty sitting or standing for long hours
   - Difficulty with relationships with boss and/or co-workers
   - Other
   - Psoriatic arthritis has no effect on my work

**[Next screen]**

**[Ask 24a to those responding that they are not currently working in Q5]**

24a. You noted that you are not currently working. Is this because of your psoriatic arthritis?

- - Yes, it’s completely related
  - Yes, it’s partially related
  - No

**[Next screen]**

**[Ask Q25 only to those who have indicated that they are a student in Q5]**

1. **At school**, do you experience any of the following because of your psoriatic arthritis? Please select all that apply.
   - Had to switch schools/programs
   - Difficulty focusing while in class or doing school work
   - Difficulty prioritizing tasks
   - My performance is affected
   - Difficulty taking tests within the allotted time
   - Takes a long time to complete required tasks
   - Difficulty being on time to class
   - Difficulty getting around campus
   - Difficulty sitting or standing in class
   - Other
   - I don’t have any difficulties at school because of my psoriatic arthritis

**[Next screen]**

1. Do you experience any of the following difficulties in your **relationships with others** because of your psoriatic arthritis? Please select all that apply.
   - Difficulty spending time with family
   - Difficulty spending time with friends
   - Difficulty maintaining my relationship with my spouse/partner
   - I have separated or divorced as a result of my psoriatic arthritis
   - Difficulty maintaining friendships
   - Lack of understanding from family and friends about my psoriatic arthritis
   - Lack of understanding from boss and/or co-workers about my psoriatic arthritis
   - Lack of support from my family and/or friends
   - Other
   - I don’t have any difficulties with my relationships because of my psoriatic arthritis

**[Next screen]**

**Section IV: Treating Your Psoriatic Arthritis**

In this next section, we’ll ask you some additional questions related to treating your psoriatic arthritis.

**[Next screen]**

1. Please select all of the **prescription** medications that you **have ever taken (currently or in the past)** for your psoriatic arthritis? Please select all that apply. **[insert drop down list of medications]**

- I’ve never taken any **prescription medications** for my condition
- A biologic medication given by injection (for example, adalimumab/Humira^®^, golimumab/Simponi^®^, etanercept/Enbrel^®^, and certolizumab/Cimzia^®^)
- A biologic medication given by infusion (for example, infliximab/Remicade^®^)
- A non-biologic disease modifying medication (for example, methotrexate, apremilast/Otezla^®^, hydroxychloroquine/Plaquenil^®^, sulfasalazine/Azulfidine^®^, and leflunomide/Arava^®^)
- Antidepressants (for example, duloxetine/Cymbalta^®^ and amitriptyline/Elavil^®^)
- A steroid medication (for example, prednisone)
- A prescription NSAID (for example, ibuprofen, naproxen, celecoxib/Celebrex^®^, meloxicam/Mobic^®^, and diclofenac/Voltaren^®^)
- Opioid pain medications (for example, Percocet^®^, morphine, and oxycodone)
- Other pain medications (for example, tramadol and gabapentin)
- Prescription sleep medications (for example, zolpidem/Ambien^®^)
- Other prescription medication

**[Next screen]**

27a. Please select all of the **over-the-counter (non-prescription)** medications that you **have ever taken (currently or in the past)** for your psoriatic arthritis? Please select all that apply. **[insert drop down list of medications]**

- I’ve never taken any **over-the-counter (non-prescription)** medications for my condition
- An over-the-counter (non-prescription) NSAID (such as aspirin, ibuprofen/Motrin^®^/Advil^®^ and naproxen/Aleve^®^ )
- Acetaminophen/Tylenol^®^
- Topical lidocaine
- Muscle relaxers
- Non-prescription sleep medications (For example, ZzzQuil^®^ and TylenolPM^®^)
- Other

**[Next screen]**

27b. Please select all of the **supplements and homeopathic** medications that you **have ever taken (currently or in the past)** for your psoriatic arthritis? Please select all that apply. **[insert drop down list of medications]**

- I’ve never taken any **supplements or homeopathic** medications for my condition
- Multi-vitamin
- Fish oil
- Vitamin D-3
- Turmeric
- Glucosamine chondroitin
- St. John’s Wort
- Essential Oils
- Medical marijuana
- Other

**[Next screen]**

**[If respondent has never taken any medication, SKIP to Q34a]**

**[Ask Q28 only to those who have taken PRESCRIPTION medications. If on OTC/Supplements, skip to Q29.]**

1. What are the reasons you have ever stopped taking or switched a **prescription** medication? Please select all that apply.
   - I have never stopped taking or switched a prescription medication
   - I didn’t like the side effects and asked for a new treatment
   - My psoriatic arthritis healthcare provider decided to give me a new treatment because of the side effects I was having
   - The medication was not controlling my psoriatic arthritis symptoms as well as I wanted it to
   - It was too expensive
   - My insurance stopped covering the medication
   - Other

**[Next screen]**

**[Q29 & Q30 Should be asked of all respondents who have ever taken any medication]**

1. How many medications do you take **in total** to treat your psoriatic arthritis? Include both prescription and non-prescription medications and supplements.

____ medications **[insert drop down; 0-99]**

**[Next screen]**

1. When deciding how to manage **your** psoriatic arthritis, which of the following describes **how** your healthcare provider discussed your treatment options with you? Please select all that apply.

- My healthcare provider presented several different options for treatment and discussed each one with me, ultimately letting me decide which treatment I would take
- I felt like my healthcare provider and I made a plan for my treatment as a team
- My healthcare provider told me my options for treatment and then provided a recommendation
- My healthcare provider told me what treatment I should take without much discussion
- I suggested a specific treatment to my healthcare provider
- Other

**[Next screen]**

**[Ask Q31-33 only to those who have ever taken PRESCRIPTION medications. Those who have only taken OTC/Supplements, skip to Q34a]**

1. How important were the following factors when choosing your **current** psoriatic arthritis **prescription** medication(s) (or most recent if not currently on prescription medication)? Please select the **top 3 factors** and rank them in order of importance, with number 1 being the most important.

**[Allow respondent to select only 3 and rank in order]**

- How well I thought I could follow the medication schedule
- Mode of administration (for example, a pill, an injection, or an infusion)
- Time to receive insurance authorization
- Whether or not my insurance covered the medication
- Total cost of the medication
- Out-of-pocket cost of the medication
- The type and severity of symptoms that I was experiencing
- How quickly the medication would start to work
- The type of side effects I might experience
- My doctor’s recommendation
- Things I’ve heard from peers or read about different psoriatic arthritis medications
- Other

**[Next screen]**

1. Sometimes patients miss a dose of their medication. What are the reasons that have caused you to miss a dose of your psoriatic arthritis **prescription** medication in the past? Please select all that apply.

- I couldn’t get to the clinic/hospital to get my infusion
- I forgot to take it
- I ran out of medication
- I wasn’t able to afford my medication
- I wanted to avoid side effects
- I didn’t have my medication with me at the time of my dose
- Insurance didn’t approve my medication in time
- The pharmacy didn’t ship my medication in time
- The doctor’s office didn’t respond to the request for refill, leading to a delay in getting my medication
- I was sick
- I was prepping for surgery
- Other
- I have never missed a dose of my psoriatic arthritis medication **[Exclusive choice]**

**[Next screen]**

1. Do you ever plan your activities around your psoriatic arthritis **prescription** medication wearing off? If yes, what activities do you plan around your medication wearing off? Please select all that apply.

- Recreational activities
- Household chores
- Work responsibilities
- Running errands
- Doing schoolwork/homework
- Spending time with my friends/family
- Travel/vacations
- I do not need to plan my activities around my medication wearing off
- Other

**[Next screen]**

**[Show Q34 only to those who have ever taken PRESCRIPTION medication. Ask Q34a to everyone else (i.e., those on no medication at all or those who are only taking OTCs or supplements)]**

1. Have you experienced any of the following issues related to accessing care for your psoriatic arthritis? Please select all that apply.

- The out-of-pocket costs for visits to my psoriatic arthritis healthcare provider(s) are high
- It’s hard to get an appointment with my psoriatic arthritis healthcare provider(s)
- It’s difficult for me to travel to my appointments with my psoriatic arthritis healthcare provider(s)
- It’s difficult to take off school/work to get to appointments with my psoriatic arthritis healthcare provider(s)
- The out-of-pocket costs for my psoriatic arthritis medication are high
- My health insurance doesn’t cover my preferred psoriatic arthritis medication
- I have difficulty filling my prescription at my preferred pharmacy
- I have difficulty getting authorization from my insurance company for my medication
- I would like to have more options for choosing a healthcare provider for my psoriatic arthritis
- Other
- I haven’t experienced any difficulties accessing care for my psoriatic arthritis **[Exclusive Choice]**

34a. Have you experienced any of the following issues related to accessing care for your psoriatic arthritis? Please select all that apply.

- The out-of-pocket costs for visits to my psoriatic arthritis healthcare provider(s) are high
- It’s hard to get an appointment with my psoriatic arthritis healthcare provider(s)
- It’s difficult for me to travel to my appointments with my psoriatic arthritis healthcare provider(s)
- It’s difficult to take off school/work to get to appointments with my psoriatic arthritis healthcare provider(s)
- I would like to have more options for choosing a healthcare provider for my psoriatic arthritis
- Other
- I haven’t experienced any difficulties accessing care for my psoriatic arthritis **[Exclusive Choice]**

**[Next screen]**

**[Repeat Q35 for each RHEUMATOLOGIST OR DERMATOLOGIST selected in SQ3]**

1. How often do you see your **[insert Rheum or Derm per SQ3**] regarding your psoriatic arthritis?

**[All options are exclusive choice]**

- Once a month
- Once every 3 months
- Once every 6 months
- Once a year
- More than once a year
- Other

**[Next screen]**

**[Repeat Q36 for each RHEUMATOLOGIST OR DERMATOLOGIST selected in SQ3]**

1. Which of the following do you regularly discuss with your **[insert Rheum or Derm per SQ3]** during visits? Please select all that apply.

- How I am feeling in general
- My psoriatic arthritis symptoms and whether they’ve changed since my last visit
- Any side effects from my psoriatic arthritis treatment
- The effect of my psoriatic arthritis on my daily activities
- The effect of my psoriatic arthritis on my life at home
- The effect of my psoriatic arthritis on my emotions
- The effect of my psoriatic arthritis on my relationships with others
- The effect of my psoriatic arthritis on work/school
- Ways I can better manage my psoriatic arthritis
- Resources I can access to help me with my psoriatic arthritis
- Other treatment options for my psoriatic arthritis
- Other

**[Next screen]**

1. What information about your psoriatic arthritis do you want to know but are unable to find?
   - Treatment options
   - Progression of disease
   - Consequences of no treatment
   - How to manage symptoms on my own
   - How to manage fatigue
   - How and when I can decrease or stop my medication
   - Patient advocacy groups or support groups available
   - Other
   - None; I have access to all of the information I need about my condition **[exclusive choice]**

**[Next screen]**

**You have reached the end of the survey.**

**Thank you for participating in this study!**
